# *Phlebotomus papatasi* sand fly predicted salivary protein diversity and immune response potential based on *in silico* prediction in Egypt and Jordan populations

**DOI:** 10.1371/journal.pntd.0007489

**Published:** 2020-07-13

**Authors:** Catherine M. Flanley, Marcelo Ramalho-Ortigao, Iliano V. Coutinho-Abreu, Rami Mukbel, Hanafi A. Hanafi, Shabaan S. El-Hossary, Emadeldin Y. Fawaz, David F. Hoel, Alexander W. Bray, Gwen Stayback, Douglas A. Shoue, Shaden Kamhawi, Scott Emrich, Mary Ann McDowell

**Affiliations:** 1 Department of Biological Sciences, Eck Institute for Global Health, University of Notre Dame, Notre Dame, Indiana, United States of America; 2 Department of Preventive Medicine and Biostatistics, F. Edward Hebert School of Medicine, Uniformed Services University of the Health Sciences, Bethesda, Maryland, United States of America; 3 Laboratory of Malaria and Vector Research, NIAID-NIH, Rockville, Maryland, United States of America; 4 Faculty of Veterinary Medicine, Jordan University of Science and Technology, Irbid, Jordan; 5 Vector Biology Research Program, U.S. Naval Medical Research Unit No. 3, Cairo, Egypt; 6 Lee County Mosquito Control District, Lehigh Acres, Florida, United States of America; 7 Min H. Kao Department of Electrical Engineering and Computer Science, University of Tennessee, Knoxville, Tennessee, United States of America; International Centre of Insect Physiology and Ecology, KENYA

## Abstract

*Phlebotomus papatasi* sand flies inject their hosts with a myriad of pharmacologically active salivary proteins to assist with blood feeding and to modulate host defenses. In addition, salivary proteins can influence cutaneous leishmaniasis disease outcome, highlighting the potential of the salivary components to be used as a vaccine. Variability of vaccine targets in natural populations influences antigen choice for vaccine development. Therefore, the objective of this study was to investigate the variability in the predicted protein sequences of nine of the most abundantly expressed salivary proteins from field populations, testing the hypothesis that salivary proteins appropriate to target for vaccination strategies will be possible. *PpSP12*, *PpSP14*, *PpSP28*, *PpSP29*, *PpSP30*, *PpSP32*, *PpSP36*, *PpSP42*, *and PpSP44* mature cDNAs from field collected *P*. *papatasi* from three distinct ecotopes in the Middle East and North Africa were amplified, sequenced, and *in silico* translated to assess the predicted amino acid variability. Two of the predicted sequences, PpSP12 and PpSP14, demonstrated low genetic variability across the three geographic isolated sand fly populations, with conserved multiple predicted MHCII epitope binding sites suggestive of their potential application in vaccination approaches. The other seven predicted salivary proteins revealed greater allelic variation across the same sand fly populations, possibly precluding their use as vaccine targets.

## Author summary

*Phlebotomus papatasi* sand flies vector *Leishmania major* parasites, one of the causative agents of cutaneous leishmaniasis (CL). Approximately 0.7–1.2 million cases of CL occur each year. CL produces disfiguring skin lesions for which no vaccine currently exists. Hematophagous arthropods secrete salivary proteins that modulate host inflammation, vasoconstriction, and blood clotting. Salivary proteins from multiple phlebotomine sand fly species have been widely studied and scrutinized to characterize their function in blood feeding as well as their ability to exacerbate or attenuate *Leishmania* infections. Hence their potential role as vaccine or as a component of vaccination strategies. In such regard, we hypothesize that in order to be successful, sand fly salivary protein-based vaccines must consider, in addition to the human immune responses, the genetic variability and the expression profiles of such salivary antigens from geographically distant sand fly populations. The purpose of this study was to analyze the genetic variability of nine of the most abundant secreted salivary proteins identified in *P*. *papatasi*, comparing insects collected from distinct ecotopes in Egypt and Jordan.

## Introduction

Leishmaniasis is a group of neglected diseases caused by *Leishmania* parasites, vectored by phlebotomine sand flies and endemic in 98 countries [1]. Different *Leishmania* species are uniquely associated with distinct clinical outcomes, ranging from cutaneous lesions to fatal visceral disease. *Leishmania major*, one of the causative agents of cutaneous leishmaniasis (CL), is transmitted by the bites of *Phlebotomus papatasi*, *P*. *bergeroti*, and *P*. *duboscqi* sand flies [2]. Approximately 0.7–1.2 million cases of CL occur each year [1]. CL produces scarring skin lesions and current treatments can be toxic, expensive, require multiple administrations, and can be difficult to access [2]. Although significant effort has been expended, there currently is no efficacious vaccine for human populations.

The salivary proteins of hematophagous arthropods are pharmacologically active molecules that modulate host inflammation, vasoconstriction, and blood clotting [3]. In *P*. *papatasi* infected with *Le*. *major*, parasites are regurgitated into the host’s skin during probing or feeding, along with a cocktail of salivary proteins, where an infection can be established. Pre-exposure to sand fly salivary proteins can exacerbate or attenuate *Leishmania* infections in animal models [4–6], and have been suggested as potential as vaccine candidates [7, 8].

It has been previously determined that exposure to uninfected *P*. *papatasi* bites confers some level of protection against *Le*. *major* in murine models, presumably via stimulation of a delayed-type hypersensitivity immune response at the site of inoculation [9]. One particular salivary protein, PpSP15, induces a Th-1 mediated immune response with a hallmark increase in IFN-γ in mice [5], and vaccination of nonhuman primates with the *P*. *duboscqi* orthologous PdSP15 resulted in a significant decrease in parasite load and lesion size, though full protection was not established [7]. Conversely, in naïve hosts, sand fly saliva exacerbates disease progression by downregulating the host’s immune response while polarizing the immune response to favor Th2 cytokine production [7, 10–12].

Genetic variability among populations of sand flies will influence the success of any salivary protein-based vaccine. Specifically, highly polymorphic salivary proteins and those under positive selection should be cautiously considered for further vaccine development. Previously, *P*. *papatasi* salivary protein 15 (PpSP15), sampled from field populations demonstrated minimal selection with a high degree of conservation at the predicted amino acid level validating PpSP15 as a potential vaccine candidate [13, 14]. Here we analyzed the genetic variability in mRNA and in the predicted peptide sequences of nine highly expressed *P*. *papatasi* salivary proteins [15, 16], including PpSP12, PpSP14, PpSP28, PpSP29, PpSP30, PpSP32, PpSP36, PpSP42, and PpSP44, from three *P*. *papatasi* populations from Egypt and Jordan.

As a geographically widespread species, *P*. *papatasi* is prevalent in the Mediterranean Basin, especially the Middle East and North Africa, with an ability to adapt to a variety of habitats that exhibit different climates, elevations, vegetation, and host species. As a result of adaptation to ecological variation, it is expected that sand flies and their salivary proteins face selective pressures that could influence vector competency and disease outcomes [17]. *P*. *papatasi* population genetics studies have demonstrated that although pockets of genetic variability exist between populations, evidence suggests that the species as a whole remains relatively homogeneous [18–23].

It remains vitally important to monitor salivary protein genetic variability to ensure the most appropriate salivary proteins are chosen as vaccine targets. A successful sand fly salivary protein-based vaccine to combat CL also depends on expression profiles and human (host) immune response to these salivary proteins, ideally selected from geographically distant sand fly populations. Egypt and Jordan are endemic for CL, with certain regions designated as hyperendemic in Jordan, with *Le*. *major* causing the majority of CL cases [24, 25]. We previously assessed the expression variability of 10 salivary gland genes [14, 26] and the genetic variability of *PpSP15* [14] of specimens inhabiting distinct ecotopes in Egypt and Jordan and throughout the sand fly season in each habitat. The purpose of this study was to analyze the genetic variability present in the nine *P*. *papatasi* salivary genes from the previous expression study [26] as potential vaccine targets that are conserved across populations and demonstrate the potential for the proteins to elicit an immune response, similar to what we reported for PpSP15 [14]. We recommend that PpSP12 and PpSP14 be considered for further vaccine development as we demonstrate that they are conserved across populations while also have potential to elicit an immune response. We caution of the use of a highly variable protein like PpSP28 for further development.

## Methods

### Sand fly collections

**Fig 1:**
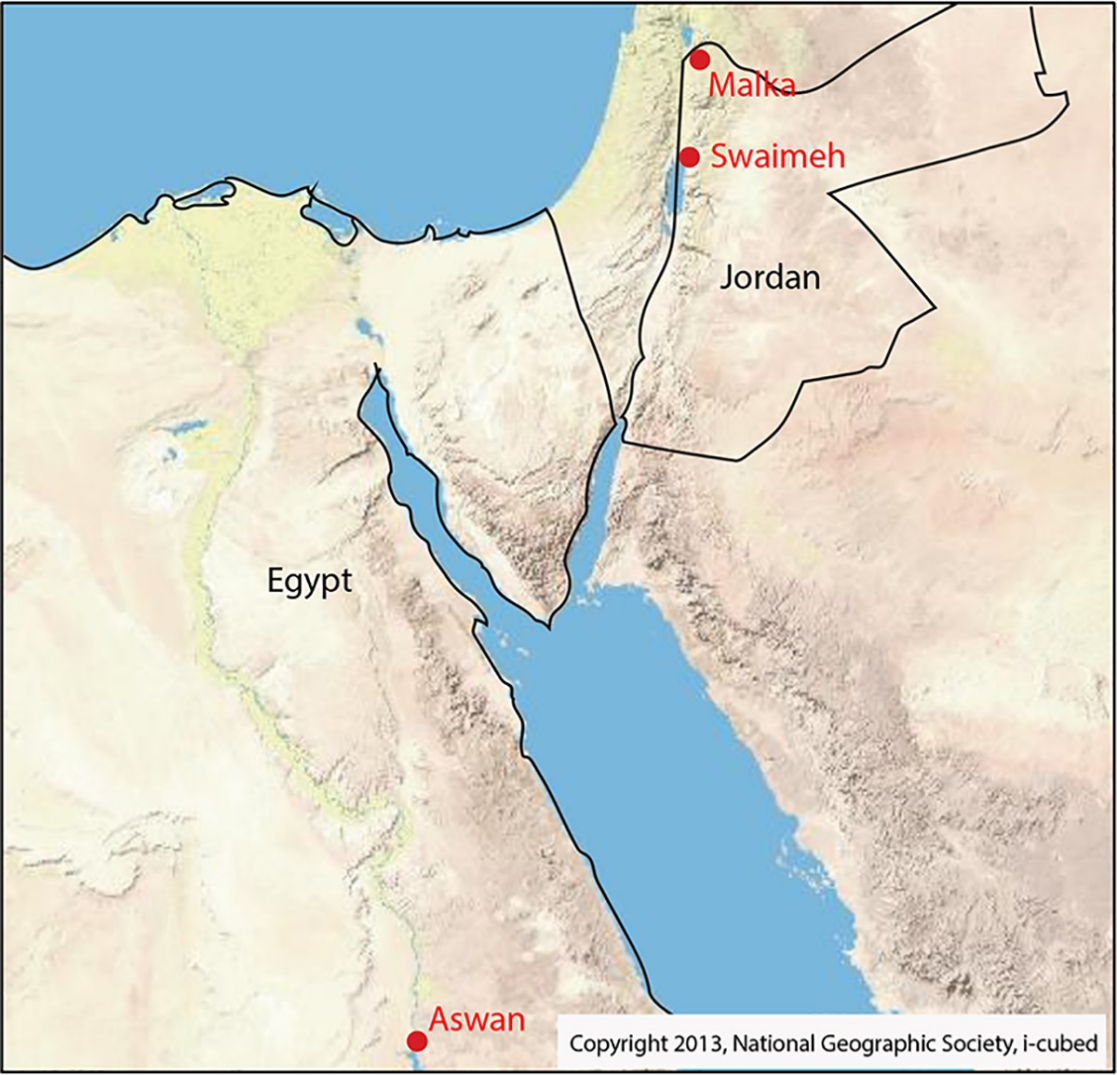
*Phlebotomus papatasi* study collection sites. Fig 1 was created using ArcMap [29].

*P*. *papatasi* were collected from one field site in each of the following locations: Aswan, Egypt (GPS coordinates N 24°10’, E 32°52’), Malka, Jordan (GPS coordinates 31°48’, E 35°35’), and Swaimeh, Jordan (N 32°40’, E 35°45’), in 2006 and 2007 (Fig 1). Both CO_2_ baited (Aswan) and non-baited (Malka and Swaimeh) CDC-style light traps collected sand flies between the hours of 18:00 and 06:00. Three trappings were attempted each year in 2006 and 2007: early (June), middle (August), and late (September). One collection occurred in Malka in late (September) 2006 while three collections occurred in Swaimeh and Aswan in late (September) 2006, early (June), and middle (August) 2007. Sand flies remained alive until dissection and were euthanized in soapy water. Flies were individually identified by microscopic examination of female spermathecae according to Lane [27], and only non-parous females were used in the analysis presented. Parity was assessed according to Anez [28]. Map in Fig 1 was created using ArcMap 10.5.1 (Redlands, CA) [29].

*P*. *papatasi* from Aswan (PPAW) were collected from a small village near the Nile River that permits artificial irrigation for the cultivation of crops like corn (*Zea mays*), wheat (*Triticum aestivum*), mangoes (*Mangifera indica*), and date palms (*Phoenix dactylifera*). Dogs, goats, and cattle are kept and raised in the village as well. The village sits at 117 m above sea level. Temperatures typically fall between 24° C and 45° C with minimal rainfall. Sand flies are abundant in this village though *Le*. *major* is absent [30]. *P*. *papatasi* collected from Swaimeh (PPJS) inhabit an area endemic for zoonotic *Le*. *major* due to the presence of *Psammomys obesus*, the reservoir host [31]. This low elevation area (~350m below sea level) experiences a Saharan Mediterranean climate with rainfall less than 50mm that occurs from November to April. Temperatures maximally range from 35–40° C in summer months and minimally range from 8–12° C in the winter. The sandy, rocky, salty soil supports halophytic and tropical flora species such as chenopods [32]. *P*. *papatasi* collected from Malka (PPJM) inhabit a rocky landscape with a typical Mediterranean climate. Malka is located at an elevation of 670 m. During the collection time in 2006, only *Le*. *tropica* was present in the region and *Le*. *major* was absent hypothesized due to the absence of *Ps*. *obesus* [33].

### Sample preparation

Dissected, *P*. *papatasi* female heads with both salivary glands intact were placed in 1.5 ml centrifuge tubes with 50 μL RNA later (Ambion, Austin, TX, USA) and homogenized with an RNAse-free pestle and hand-held homogenizer. Samples were stored at 4° C for up to 48 hours, shipped on dry ice, and then stored at -80° C until analyzed. Table 1 outlines the number of individuals from each site for each salivary protein.

**Table 1:** *Phlebotomus papatasi* salivary transcript amplicon length and number of individual sand flies per collection site.

Salivary Gene	Reference Accession #	Amplicon length (bp)	% Coverage	% Similarity to Reference Gene	% Similarity to Reference Protein	All	PPAW	PPJM	PPJS
*PpSP12*	JQ988874.1	291	54	97.25–100	95.88–100	96	26	29	41
*PpSP14*	JQ988880.1	246	49	97.56–100	95.12–100	119	29	44	46
*PpSP28*	AF335488.1	554	63	95.13–99.82	89.13–100	111	26	30	55
*PpSP29*	JQ988887.1	651	60	98–99.54	97.24–100	126	46	38	42
*PpSP30*	JQ988884.1	183	21.7	96.17–99.45	93.33–100	70	20	28	22
*PpSP32*	JQ988888.1	568	65	98.77–99.65	97.35–99.47	130	42	45	43
*PpSP36*	JQ988892.1	637	58	98.59–100	97.17–100	82	25	22	35
*PpSP42*	JQ988885.1	614	46	97.39–99.84	95.1–100	109	27	45	37
*PpSP44*	JQ988886.1	675	51	98.52–99.85	98.52–99.85	121	35	44	42

Amplicon lengths = base pairs. PPAW: Aswan, Egypt; PPJM: Malka, Jordan; PPJS: Swaimeh, Jordan.

### RNA extraction and cDNA synthesis

Total RNA was extracted from the heads and salivary glands of individual *P*. *papatasi* samples using the RNeasy Mini RNA isolation kit (Qiagen, Valencia, CA, USA). cDNAs were synthesized using Invitrogen reagents (Invitrogen, Carlsbad, CA, USA), per manufacturer’s specifications as we previously performed [14, 26].

### Sequence analyses

cDNAs produced from the total RNA of individual *P*. *papatasi* were amplified by PCR. Primers used to amplify each salivary transcript can be found in S1 Table. PCR products were purified by twice washing in 150 μL DNAse/RNAse-free water (Invitrogen, Carlsbad, CA, USA) in Multiscreen PCR cleaning plates (Millipore, Burlington, Massachusetts, USA) with vacuum application (10 psi). Purified PCR products were resuspended in 50 μL sterile water. Leading and lagging strands were sequenced and poor-quality sequences were excluded from the analyses. Forward and reverse chromatograms were inspected and consensus sequences were aligned using MEGA [34] and manually corrected. The resulting sequences were deposited in GenBank (http://ncbi.nlm.nih.gov) and accession numbers can be found in S1 Table.

### Multi-copy assessment of salivary proteins

We looked for copy number variation relative to currently assembled *P*. *papatasi* loci by following the approach of Miles *et al*. [35]. In short, paired sequences from the two individual entries with the most reads (SRR1997534 and SRR199776) were downloaded from SRA. After initial overall quality checking using FastQC [36], these paired reads were then aligned to the Ppap reference assembly using BWA 0.5.9r16 [37]. Next, based on the resulting alignments (SAM output), reads were placed into non-overlapping 300 bp bins such that each bin contained all reads whose alignment started in its corresponding 300 bp interval. Unlike Miles *et al*. [35] there were no known “core genome” coordinates that excluded repeat regions for computing a less biased average count for normalization. Therefore, discrete counts in each bin were normalized based on the average count across all scaffolds, median count, and average count excluding terminal 2 kb of scaffolds (1 kb on 5’ and 3’ of scaffolds). Although normalization slightly differed, the results in terms of under (less than 0.5 average/median) and over (more than 2 average/median) remained the same within and between the two samples considered. Because of under assembly of heterologous regions, the final normalization value was computed based on the empirical distribution of read bin counts with the value with the greatest number of entries near the computed overall average. Significance was derived using a Poisson model parameterized with this estimate as lambda using the Lander-Waterman model of sequence sampling [38], and a Bonferroni correction was applied to correct for multiple comparisons.

### Population analyses

Both interpopulation and intrapopulation analyses were performed using DnaSP v.6 [38]. Interpopulation parameters assessed included: fixation indexes such as Fst [39, 40] and Gst [41], as well as Hs and Ks indexes [42]. Other parameters assessed included: neutral evolution hypothesis [43] and neutrality tests Tajima’s D [44] and Fu and Li’s D and F [45]. The Ka/Ks ratio (ω) for the predicted salivary protein as well as a sliding window analysis of 70 codons each was calculated.

Weblogos [46] pictorially depict the relative frequencies of polymorphic nucleotides and amino acids. The height of the bases indicate relative frequency and conservation is depicted by the overall weight of the stack. Network 5 [47] generated median joining networks exhibiting haplotype relationships.

### Secondary structure and T-cell epitope predictions

Secondary structure predictions for each salivary protein were generated using a secondary structure prediction tool (http://bioinf.cs.ucl.ac.uk/psipred) with default parameters based on the consensus sequence for all individual amino acid sequences from DnaSP. Two different predictions tools predicted the promiscuous HLA-class II binding sites and human T-cell epitopes: IEDB analysis resource T-cell epitope prediction tools (http://tools.immuneepitope.org/main/html/tcell_tools.html) [48, 49] and ProPred MHC class II binding prediction server (http://www.imtech.res.in/raghava/propred/) [50]. For the 51 HLA alleles tested in ProPred, thresholds included a promiscuous search set to 3%. For the 27 HLA alleles tested in IEDB, only predicted peptides with a Consensus percentile rank of 0.10 or below are included as the top 10% of peptides with the strongest predicted binding affinity.

## Results

Using our previously published PpSP15 data [14] as a guide, herein, we present data for PpSP12, PpSP14, and PpSP28 as a representative predicted salivary proteins with PpSP29, PpSP30, PpSP32, PpSP36, PpSP42, and PpSP44 data provided in the supplemental materials.

### PpSP12 in-depth analyses

#### Nucleotide and amino acid genetic diversity

**Fig 2:**
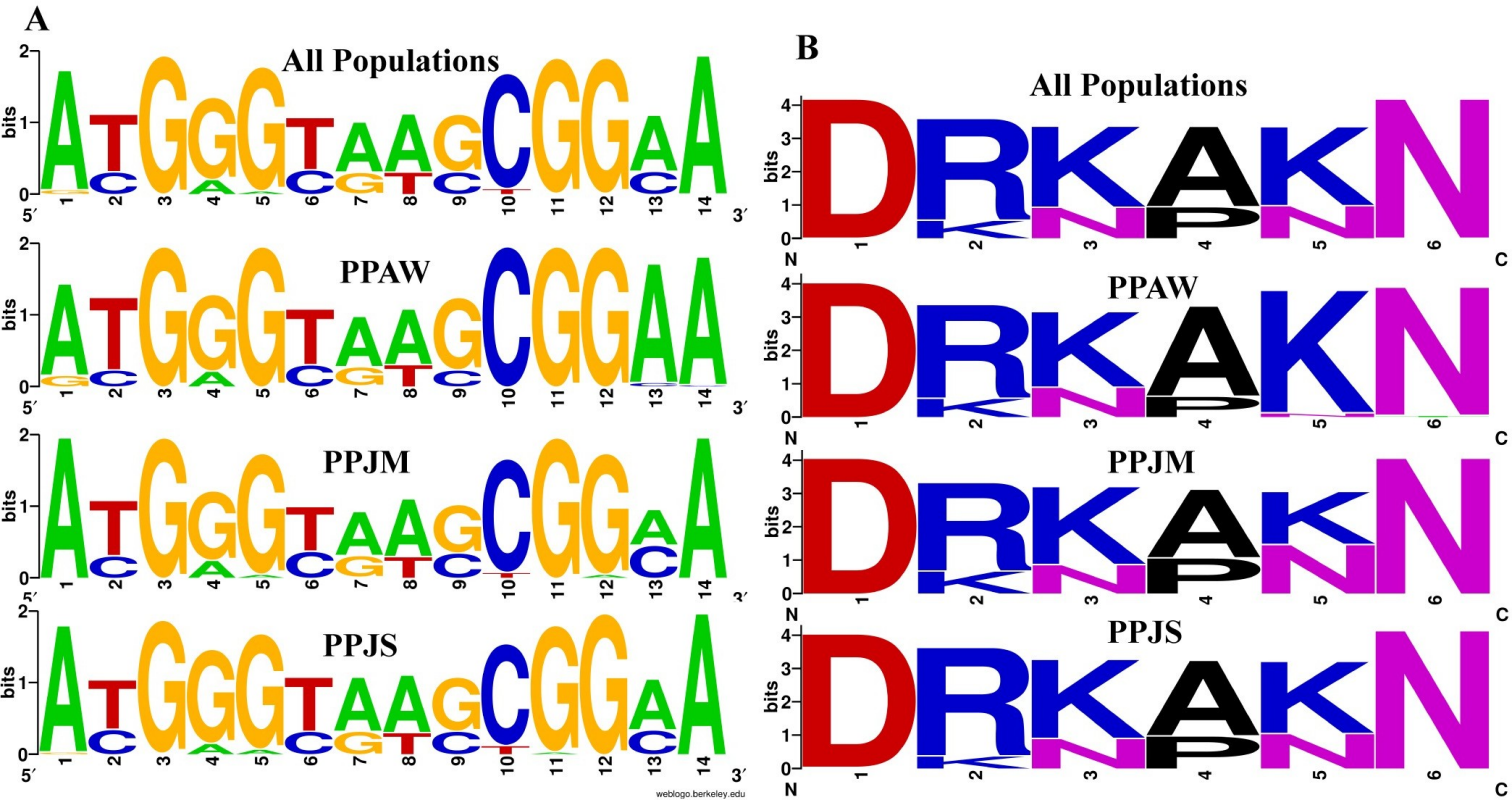
PpSP12 nucleotide and amino acid variation. (A) Weblogo illustrating the relative frequencies of nucleotide polymorphisms in wild caught *P*. *papatasi* populations from PPAW, PPJM, and PPJS. (B) Weblogo illustrating the relative frequencies of amino acid polymorphisms in wild caught *P*. *papatasi* populations from PPAW, PPJM, and PPJS.

The 291 bp *PpSP12* fragment produced 14 polymorphic sites (Fig 2A). Two specific nucleotide positions indicate limited heterogeneity between the Jordan populations compared to the Egypt population. Position one shows conservation of adenine in both Jordan populations with variation present in the Egypt population. Conversely, in position 13 the conserved frequency of adenine is greater in the Aswan population in comparison to both Malka and Swaimeh. All of the populations present similar heterogeneity at positions 6–9. Although heterogeneity exists in *PpSP12*, it is the lowest when comparing all 9 salivary proteins. The translated PpSP12 amino acid sequence has 6 variable positions out of 97 total amino acids (Fig 2B). At position 2, arginine and lysine are both found in all populations. The frequency of alanine and proline are relatively equal for all populations in position 4. Both of these amino acids are small in size, nonpolar, and hydrophobic. At position 3 and 5, the relative frequencies of lysine and asparagine are the same except the Aswan population at position 5 has a much higher frequency of lysine.

#### Population genetics analysis

**Fig 3:**
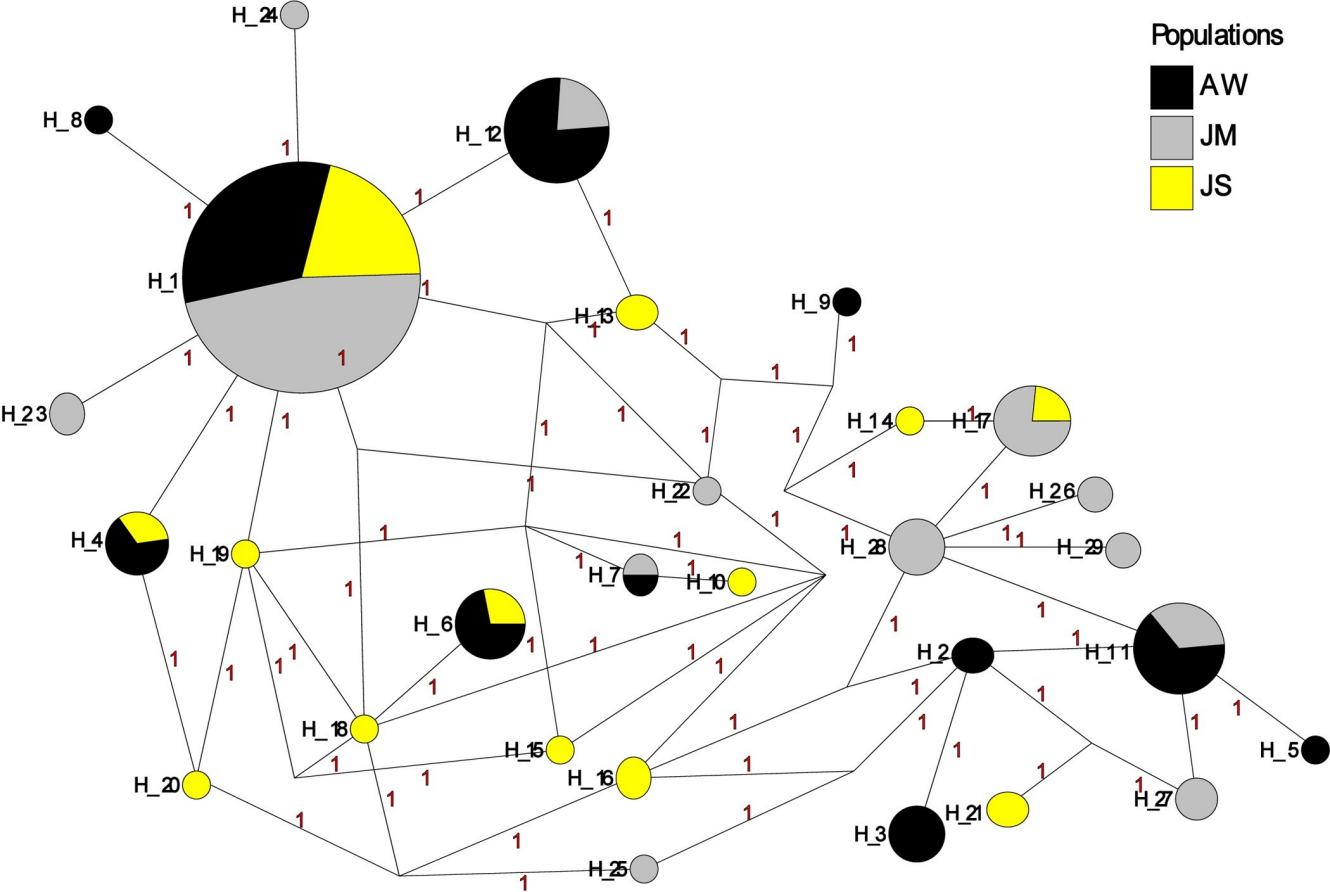
Median-joining network for PpSP12 *P*. *papatasi* haplotypes. Circle size and circle color indicates frequency and geographical location of haplotypes, respectively. Haplotype numbers are written next to the corresponding circle H_XX. Red numbers between haplotypes indicate number of mutations between haplotypes.

A total of 96 cDNA sequences were analyzed for *PpSP12* from Aswan (n = 26), Malka (n = 29), and Swaimeh (n = 41). Twenty-nine haplotypes were identified with 14 variant sites. Of the 29 haplotypes, 22 were found in only one of the geographic study sites and 6 of the 7 shared haplotypes were found in 2 populations. One haplotype, H_1, was present in all 3 populations and was the most common haplotype. The Aswan, Egypt, population had 5 unique haplotypes (H_2, H_3, H_5, H_8, H_9) with 3 of those being private haplotypes (H_5, H_8, H_9). The Malka, Jordan, population exhibited 9 unique haplotypes (H_10, H_13, H_14, H_15, H_16, H_18, H_19, H_20, H_21) with 6 of the 9 being private haplotypes (H_10, H_14, H_15, H_18, H_19, H_20). The Swaimeh, Jordan, population demonstrated 8 unique haplotypes (H_22 to H_29) with 3 private haplotypes (H_22, H_24, H_25). A variety of population genetics parameters were assessed (Table 2) indicating genetic homogeneity for *PpSP12* across the three populations. Tajima’s D and Ka/Ks analysis indicated that this protein is not undergoing positive selection but rather it is either neutral or possibly experiencing purifying selection (Table 2). Furthermore, population structure is not indicated as Fst uncovered little genetic variability in pairwise comparisons (Table 3). The *PpSP12* median-joining network does not demonstrate any notable clustering separating the different populations from one another (Fig 3). Although there are 29 total haplotypes, the haplotypes are differentiated from one another by only one mutation. The Ka/Ks ratio, a diversifying selection index, was 0.293 or less across the sliding window analysis of the protein for all populations indicating purifying or stabilizing selection of this protein (Table 4).

**Table 2:** PpSP12 population genetics analyses for *P*. *papatasi* populations.

Population	All Data	PPAW	PPJM	PPJS
Number of Sequences	96	26	29	41
Number of Sites	291	291	291	291
- Monomorphic	277	282	281	279
- Polymorphic	14	9	10	12
Singleton variable sites	2	1	0	1
- Site positions	64, 260	260	-	64
Parsimony informative sites	11	8	10	11
- Site positions	36, 54, 74, 75, 108, 117, 124, 126, 138, 150, 252	36, 54, 74, 108, 114, 117, 124, 252	54, 74, 75, 108, 114, 117, 124, 126, 150, 252	36, 54, 74, 75, 108, 114, 117, 124, 126, 138, 252
Segregating sites (S)	14	9	10	12
Total number of mutations (Eta)	16	10	11	12
Total number of synonymous changes	10	5	7	7
- Site positions	36, 54, 75, 108, 114, 114, 114, 126, 138, 150,	36, 54, 108, 114, 114	54, 75, 108, 114, 114, 126, 150	6, 54, 75, 108, 114, 126, 138
Total number of replacement changes	6	5	4	5
- Site positions	64, 74, 117, 124, 252, 260	74, 117, 124, 252, 260	74, 117, 124, 252	64, 74, 117, 124, 252
Number of haplotypes	29	9	14	14
Haplotype diversity (Hd)	0.723	0.58145	0.81307	0.68473
- Standard deviation of Hd	0.034	0.076	0.034	0.055
Nucleotide diversity (Pi)	0.01063	0.00864	0.01139	0.01089
Standard deviation of Pi	0.00067	0.00135	0.00105	0.00102
Theta (per site) from Eta	0.00943	0.00760	0.00817	0.00828
Theta (per site) from S (Theta-W)	0.00825	0.00684	0.00742	0.00828
- Standard deviation of theta (no recombination)	0.00279	0.00288	0.00300	0.00310
- Standard deviation of theta (free recombination)	0.00220	0.00228	0.00235	0.00239
Theta (per site) from Pi	0.01078	0.00874	0.01157	0.01105
Average number of nucleotide differences (k)	3.094	2.51357	3.31579	3.16893
Theta estimated from Eta	2.743	2.21297	2.376	2.411
Fu and Li’s D test statistic	-0.72468	0.15876	0.84321	0.86940
Statistical significance	NS	NS	NS	NS
Fu and Li’s F test statistic	-0.38441	0.27545	1.10728	1.02795
- Statistical significance	NS	NS	NS	NS
Tajima’s D	0.33033	0.38568	1.12200	0.85937
- Statistical significance	NS	NS	NS	NS
Synonymous sites Tajima’s D(Syn)	-0.19687	0.57799	0.16187	0.43992
Statistical significance	NS	NS	NS	NS
Nonsynonymous sites Tajima’s D(Nonsyn)	0.98069	0.07503	2.14787	1.10242
- Statistical significance	NS	NS	NS	NS
Silent sites Tajima’s D(Sil)	-0.19687	0.57799	0.16187	0.43992
- Statistical significance	NS	NS	NS	NS
Tajima’s D (Nonsyn/Syn) ratio	-4.98139	0.12982	13.26885	2.50597
ω (Ka/Ks)	---	0.222	0.284	0.242

NS = *p*>0.10; NS^1^ = 0.10 > *p* > 0.05; * = *p*<0.05

**Table 3:** *PpSP12* pairwise comparisons of genetic differentiation estimates.

POP 1	POP 2	Hs	Ks	Gst	Fst	Dxy	Da
PPAW	PPJM	0.70381	2.93656	0.05362	0.06720	0.01074	0.00072
PPAW	PPJS	0.64501	2.91461	0.01242	0.03412	0.01011	0.00034
PPJM	PPJS	0.73758	3.22977	0.02492	-0.00011	0.01114	0.00000

**Table 4:** PpSP12 sliding window analysis.

	Ka/Ks		
Sliding Window	PPAW	PPJM	PPJS
1–70	0.000	0.000	0.013
71–140	0.293	0.256	0.213
141–210	0.000	0.000	0.000
211–280	----	----	----
281–291	0.000	0.000	0.000

Ka/Ks were plotted for every 70 codons. Values greater than one suggest the potential for positive selection. ----indicates a lack of polymorphic data in the window to calculate a Ka/Ks value.

#### Secondary structure & T-cell epitope predictions

**Fig 4:**
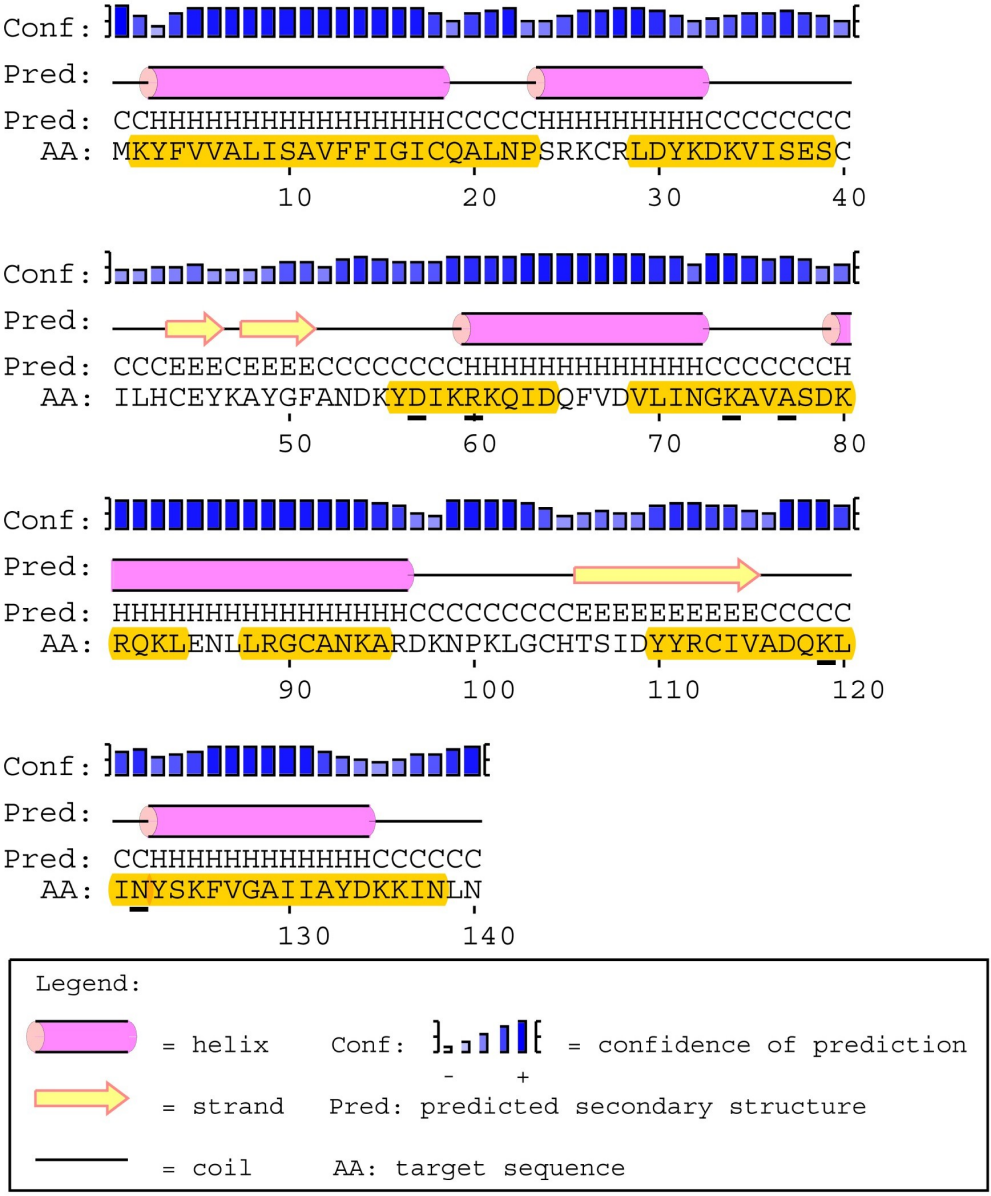
PpSp12 secondary structure, polymorphic sites, and MHC class II epitope predictions. The mature PpSP12 amino acid sequence predicted secondary structure. Yellow highlighted amino acids indicate the predicted MHC class II predicted promiscuous peptides. Individual amino acids underlined in black indicate unique polymorphic sites. Predicted secondary structure based on sequence accession #AGE83083 [51].

The predicted amino acid sequence for PpSP12 exhibited only one polymorphic site (R60) that was found in an α-helix whereas the other six polymorphic sites were found in predicted coils (D57, K74, A77, K119, N122) (Fig 4). All 6 polymorphic sites are found in predicted MHC II T-cell epitope binding sites though this should not interfere with the potential for T-cell activation as the polymorphic sites are found in the middle of the predicted binding sites and surrounded by conserved regions. Of the 140 amino acids included in this analysis, 95 amino acids were predicted to be potential epitope recognition sites. The areas of the amino acid sequence with the highest predicted binding affinities occur between the lysine residue at position 2 (K2) and the proline residue at position 23 (P23) as well as the tyrosine residue at position 110 (Y110) and the asparagine residue at position 138 (N138). Of the 78 total HLA alleles tested using the two software tools, all 51 alleles from ProPred identified potential binding sites though certain alleles, such as DRB1_03, DRB1_11, and DRB1_13, had greater predicted binding affinities than the others. The alleles with the strongest binding affinity potential identified by IEDB software included DQA1_0401/DQB1_0402, DPA1_0103/DPB1_0201, and DRB1_0301. The DQA1/DQB1 and DPA1/DPB1 alleles demonstrated a greater affinity for residues between K2 to A20 and DRB1_0301 demonstrated a greater affinity for Y110 to F126, bookending the mature PpSP12.

### PpSP14 in-depth analyses

#### Nucleotide and amino acid genetic diversity

**Fig 5:**
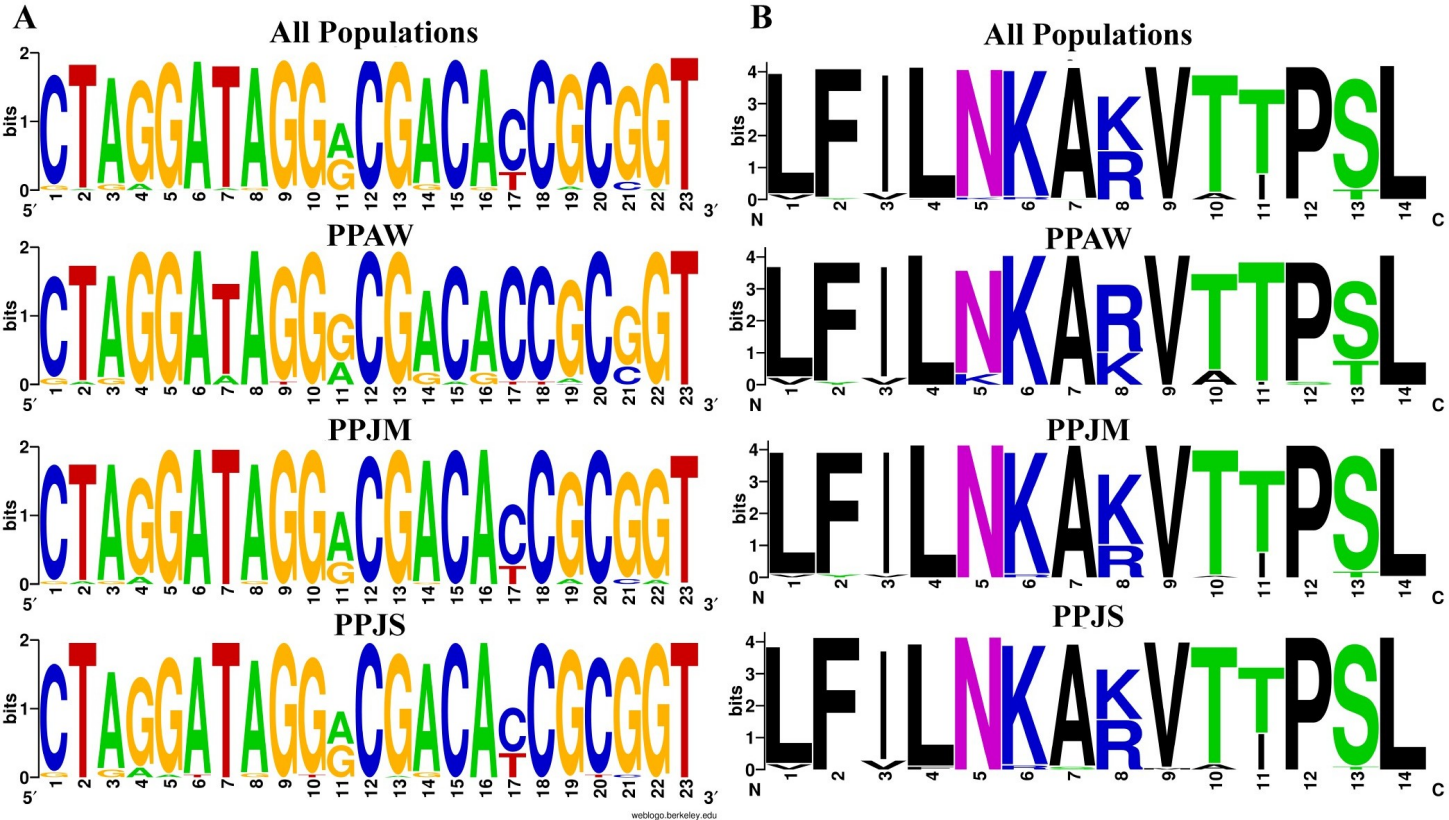
PpSP14 nucleotide and amino acid variation. (A) Weblogo illustrating the relative frequencies of nucleotide polymorphisms in wild caught *P*. *papatasi* populations from PPAW, PPJM, and PPJS. (B) Weblogo illustrating the relative frequencies of amino acid polymorphisms in wild caught *P*. *papatasi* populations from PPAW, PPJM, and PPJS.

The 246-bp *PpSP14* fragment produced 23 polymorphic sites (Fig 5A). Similar to *PpSP12*, there exists limited heterogeneity between the populations studied. Position 11 demonstrates variation in all 3 populations with equal representation of adenine and guanine. In position 17, the Jordan populations have roughly equal rates of cytosine and guanine but the Egypt population has cytosine in the majority of individuals. Guanine dominates at position 21 in both Jordan populations but is equally represented with cytosine in the Egypt population. The remaining 20 polymorphic sites present similar levels of heterogeneity across all 3 populations. The translated PpSP14 amino acid sequence has 14 variable positions out of 82 total amino acids (Fig 5B). At position 1 and 3, leucine, valine, and isoleucine are all easily substituted for one another since they are hydrophobic and prefer to be buried in the protein core. In position 5, the Aswan, Egypt, population demonstrates limited substitution of the asparagine amino acid with lysine, both polar amino acids. Lysine and arginine are relatively equal for all populations at position 8. Serine and threonine were present at position 13, but more threonine is found in the Egypt population compared to the Jordan populations. At position 10, threonine and alanine substitutions are found in all populations but are more frequent in the Egypt population. At position 11, threonine is substituted with isoleucine in both Jordanian populations.

#### Population genetics analysis

**Fig 6:**
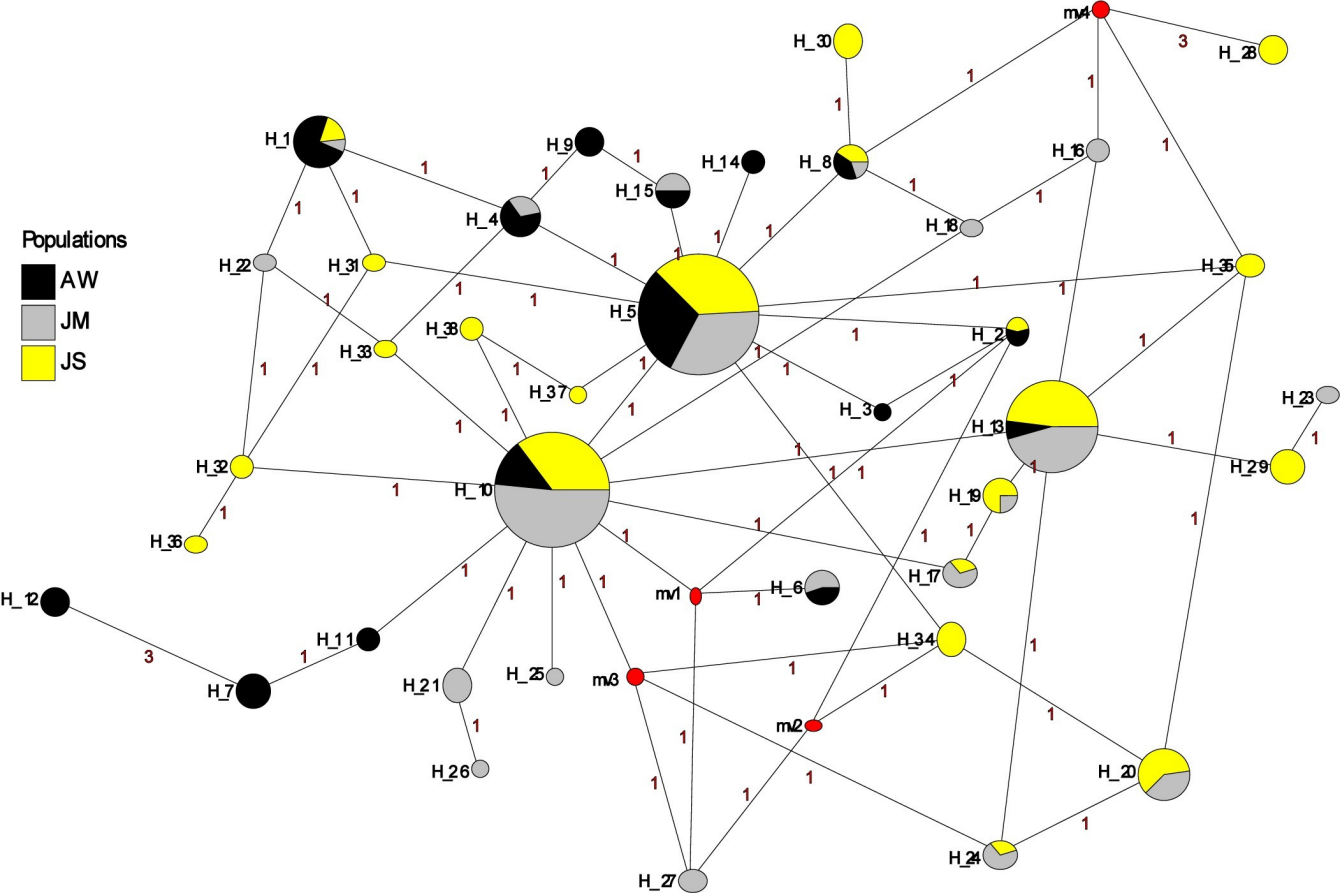
Median-joining network for PpSP14 *P*. *papatasi* haplotypes. Circle size and circle color indicates frequency and geographical location of haplotypes, respectively. Haplotype numbers are indicated next to the corresponding circle H_XX. Red numbers between haplotypes indicate number of mutations between haplotypes.

A total of 119 cDNA sequences were analyzed for *PpSP14* from PPAW (n = 29), PPJM (n = 44), and PPJS (n = 46). Thirty-eight haplotypes were identified with 23 variant sites. Of the 38 haplotypes, 25 were found in only one of the geographic study sites, 8 were shared between PPAW/PPJM or PPJM/PPJS, but none were shared by PPAW/PPJS. Five haplotypes were present in all three populations (H_1, H_5, H_8, H_10, H_13), with H_5 the most common haplotype. PPAW had 6 unique haplotypes (H_3, H_7, H_9, H_11, H_12, H_14) with 1 of those designated a private haplotype (H_3). PPJM had 8 unique haplotypes (H_16, H_18, H_21, H_22, H_23, H_25, H_26, H_27) with 6 of the 8 being private haplotypes (H_16, H_18, H_22, H_23, H_25, H_26). PPJS had 11 unique haplotypes (H_28, H_29, H_30, H_31, H_32, H_33, H_34, H_35, H_36, H_37, H_38) with 6 of the 11 being private haplotypes (H_31, H_32, H_33, H_36, H_37, H_38). The population genetics assessment indicates genetic homogeneity for *PpSP14* across the 3 populations (Table 5). Although the Tajima’s D values were negative across all populations, the values were not significant and do not deviate far from zero indicating no selection. The majority of Ka/Ks values are under 1 or do not deviate far from 1 further indicating no selection acting on *PpSP14*. There is the potential for population structuring as Fst demonstrated moderate genetic differentiation between PPAW and PPJM (0.10771) and PPAW and PPJS (0.09091) and little genetic differentiation between PPJM and PPJS (0.02346) (Table 6). The *PpSP14* median joining network does not demonstrate any significant clustering separating the different populations from one another (Fig 6). The PPJS haplotypes might be clustering together as compared to PPAW and PPJM but the 38 haplotypes are differentiated from one another by only one mutation. The only exception being haplotypes H_7 and H_12, both from PPAW are differentiated by 3 mutations from one another. The Ka/Ks ratio in the sliding window from 141–210 indicated potential positive selection in both PPJM and PPJS populations but not in the PPAW population (Table 7).

**Table 5:** PpSP14 population genetics analyses for *P*. *papatasi* populations.

Population	All Data	PPAW	PPJM	PPJS
Number of Sequences	119	29	44	46
Number of Sites	246	246	246	246
- Monomorphic	223	233	233	232
- Polymorphic	23	13	13	14
Singleton variable sites	2	0	2	1
- Site positions	147, 232	-	147, 232	183
Parsimony informative sites	21	13	11	13
Site positions	1, 14, 49, 54, 69, 75, 99, 116, 129, 133, 143, 151, 154, 156, 162, 179, 181, 183, 216, 221, 231	1, 14, 49, 99, 129, 143, 154, 156, 162, 179, 181, 183, 221	1, 14, 49, 54, 116, 143, 154, 179, 183, 221, 231	1, 49, 54, 69, 75, 116, 133, 143, 151, 154, 179, 216, 221
Segregating sites (S)	23	13	13	14
Total number of mutations (Eta)	24	14	13	14
Total number of synonymous changes	8	3	4	4
- Site positions	54, 69, 129, 147, 156, 162, 216, 231	129, 156, 162	54, 147, 183, 231	54, 69, 183, 216
Total number of replacement changes	13	8	9	10
- Site positions	1, 14, 49, 75, 99, 116, 133, 143, 151, 154, 179, 221, 232	1, 14, 49, 99, 143, 154, 179, 221	1, 14, 49, 116, 143, 154, 179, 221, 232	1, 49, 75, 116, 133, 143, 151, 154, 179, 221
Number of haplotypes	38	15	20	21
Haplotype diversity (Hd)	0.870	0.876	0.833	0.874
- Standard deviation of Hd	0.013	0.029	0.026	0.019
Nucleotide diversity (Pi)	0.00817	0.00905	0.00657	0.00814
- Standard deviation of Pi	0.00046	0.00102	0.00056	0.00070
Theta (per site) from S (Theta-W)	0.01546	0.01142	0.01047	0.01117
- Standard deviation of theta (no recombination)	0.00450	0.00430	0.00381	0.00397
- Standard deviation of theta (free recombination)	0.00322	0.00317	0.00290	0.00299
Theta (per site) from Pi	0.00825	0.00916	0.00663	0.00823
Average number of nucleotide differences (k)	2.009	2.226	1.616	2.003
Theta estimated from Eta	3.969	3.024	2.575	2.749
Fu and Li’s D test statistic	0.46088	1.04412	0.35095	0.99210
- Statistical significance	NS	NS	NS	NS
Fu and Li’s F test statistic	-0.32793	0.49487	-0.16267	0.43593
- Statistical significance	NS	NS	NS	NS
Tajima’s D	-1.33534	-0.78153	-1.02243	-0.75052
- Statistical significance	NS	NS	NS	NS
Synonymous sites Tajima’s D(Syn)	-1.63036	-0.83456	-1.08859	-1.12766
- Statistical significance	NS^1^	NS	NS	NS
Nonsynonymous sites Tajima’s D(Nonsyn)	-0.63507	-0.12244	-0.76166	-0.40308
- Statistical significance	NS	NS	NS	NS
Silent sites Tajima’s D(Sil)	-1.6306	-0.83456	-1.08859	-1.12766
- Statistical significance	NS^1^	NS	NS	NS
Tajima’s D (Nonsyn/Syn) ration	0.38953	0.14672	0.69967	0.35745
ω (Ka/Ks)	---	0.877	0.879	1.242

NS = *p*>0.10; NS^1^ = 0.10 > *p* > 0.05; * = *p*<0.05

**Table 6:** *PpSP14* pairwise comparisons of genetic differentiation estimates.

POP 1	POP 2	Hs	Ks	Gst	Fst	Dxy	Da
PPAW	PPJM	0.84968	1.85842	0.01880	0.10771	0.00875	0.00094
PPAW	PPJS	0.87453	2.08910	001161	0.09091	0.00945	0.00086
PPJM	PPJS	0.85355	1.81360	0.00205	0.02346	0.00753	0.00018

**Table 7:** PpSP14 sliding window analysis.

	Ka/Ks		
Sliding Window	PPAW	PPJM	PPJS
1–70	----	0.322	0.242
71–140	0.748	----	----
141–210	0.541	1.868	14.944
211–246	----	0.330	0.220

Ka/Ks were plotted for every 70 codons. Values greater than one suggest the potential for positive selection. ----indicates a lack of polymorphic data in the window to calculate a Ka/Ks value.

#### Secondary structure & T-cell epitope predictions

**Fig 7:**
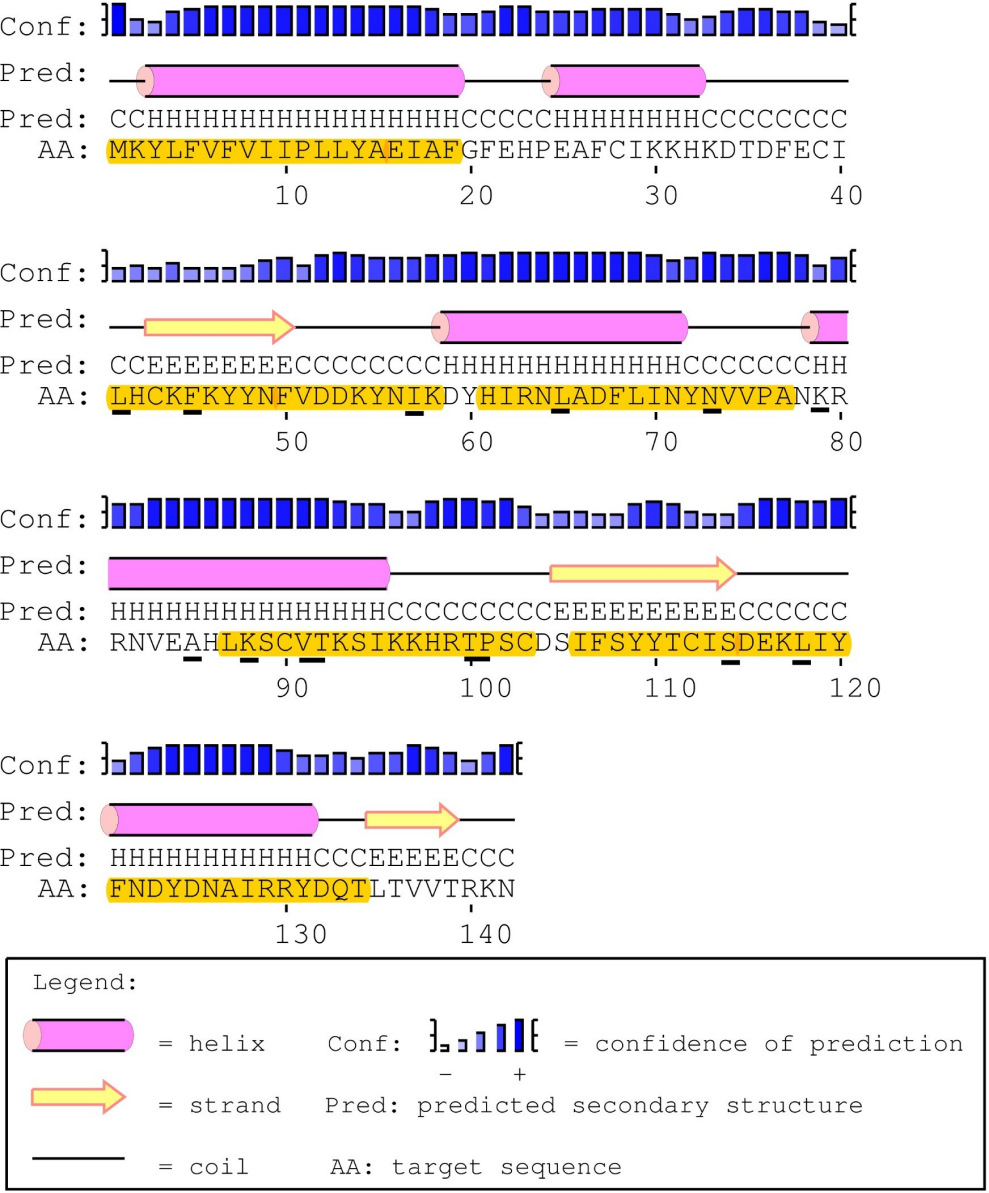
PpSp14 secondary structure, polymorphic sites, and MHC class II epitope predictions. The mature PpSP14 amino acid sequence predicted secondary structure. Yellow highlighted amino acids indicate the predicted MHC class II predicted promiscuous peptides. Individual amino acids underlined in black indicate unique polymorphic sites. Predicted secondary structure based on sequence accession #AGE83089 [51].

The predicted amino acid sequence for PpSP14 has 6 polymorphic sites (L65, K79, A85, K88, V91, T92) that were predicted to be in α-helices whereas the other 8 polymorphic sites were found in predicted coils (L41, F45, I57, N73, T100, P101, S114, L118) (Fig 7). Twelve of the 14 polymorphic sites were found in predicted MHC II T-cell epitope binding sites. This variability may affect the predicted binding sites found between amino acids L41 and K58 and between amino acids L87 and C103, as the variable sites were found at the beginning and end of the fragment. There are no polymorphic sites found between amino acids M1 and F19. The variable sites found between H61 and A77 and I106 and T134 were found in the middle of the amino acid fragment and should not hinder binding. Of the 142 amino acids included in this analysis, 100 amino acids were predicted to be potential epitope recognition sites. The software prediction tools, IEDB and ProPred, agree that the areas of the amino acid sequence with the highest predicted binding affinities occur between methionine residue at position 1 (M1) and the phenylalanine residue at position 19 (F19) as well as the isoleucine residue at position 106 (I106) and the threonine residue at position 134 (T134). Similar to PpSP12, all 51 alleles from ProPred identified potential binding sites, particularly between residues M1 and F19. Alleles DRB1_04XX, DRB1_08XX, DRB1_11XX, DRB1_13XX, and DRB1_15XX had the highest binding affinities overall. To a lesser extent, the following alleles were also identified DRB1_010X, DRB1_030X, DRB1_070X, and DRB5_010X. The alleles with the strongest binding affinity potential identified by IEDB software included DRB3_0101, DPA1_0301/DPB1_0402, DRB1_1101, DAQ1_0101/DQB1_0501, DPA1_0103/DPB1_0201, DRB1_0301, DPA1_0201/DPB1_0101, and DRB5_0101.

### PpSP28 In-depth Analyses

#### Nucleotide and amino acid genetic diversity

**Fig 8:**
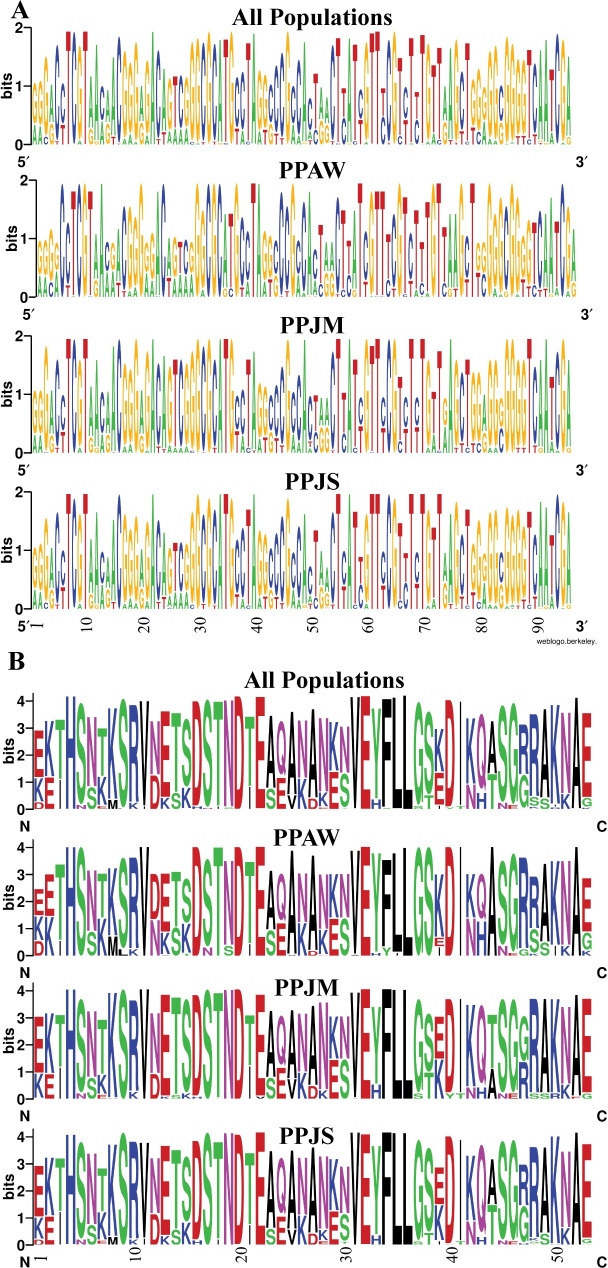
PpSP28 nucleotide and amino acid variation. (A) Weblogo illustrating the relative frequencies of nucleotide polymorphisms in wild caught *P*. *papatasi* populations from PPAW, PPJM, and PPJS. (B) Weblogo illustrating the relative frequencies of amino acid polymorphisms in wild caught *P*. *papatasi* populations from PPAW, PPJM, and PPJS.

The 651-bp *PpSP28* fragment produced 95 polymorphic sites (Fig 8A). Approximately, 61% of the polymorphic sites are transition substitutions and 26% are transversions. The remaining 12% of polymorphic sites are mostly conserved as the number of substitutions are so few. Positions 50 and 90 have three possible options at this site, each site a different combination of guanine, cytosine, adenine, or thymine. The translated PpSP28 amino acid sequence has 53 variable positions out of 184 total amino acids (Fig 8B). Eighteen of the variable sites demonstrate limited heterogeneity while the other 35 sites demonstrate significant variation between the populations and an abundance of amino acid substitutions. PpSP28 exhibits the greatest nucleotide and amino acid sequence variability of all 9 salivary proteins studied (Fig 8).

#### Population genetics analysis

**Fig 9:**
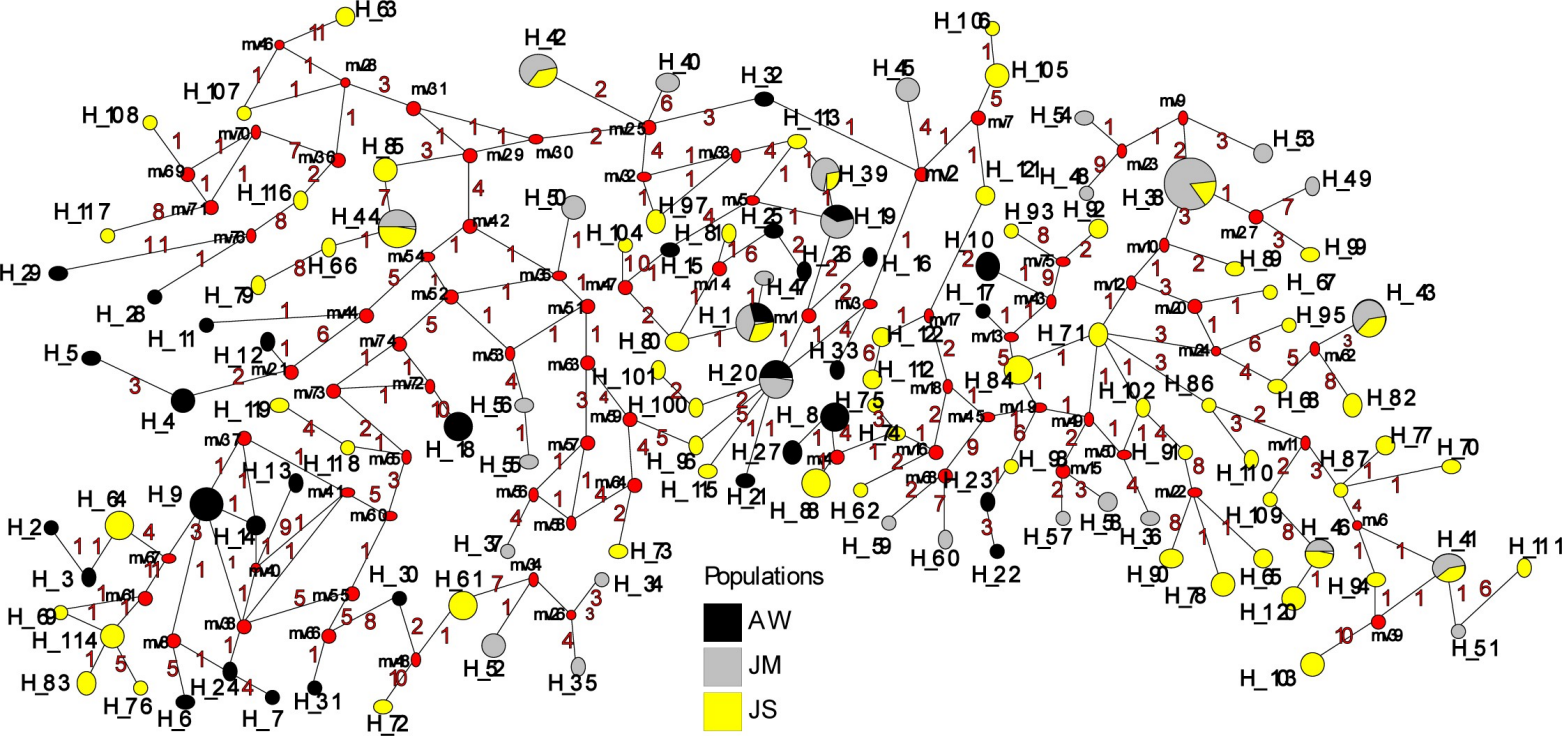
Median-joining network for PpSP28 *P*. *papatasi* haplotypes. Circle size and circle color indicates frequency and geographical location of haplotypes, respectively. Haplotype numbers are indicated next to the corresponding circle H_XX. Red numbers between haplotypes indicate number of mutations between haplotypes.

A total of 111 cDNA sequences were analyzed for *PpSP28* from Aswan (n = 26), Malka (n = 30), and Swaimeh (n = 55). Ninety-five variant sites were identified in 122 haplotypes. Swaimeh, Jordan, tallied the most unique haplotypes (62) with 46 identified as private. Malka, Jordan, had 21 unique haplotypes with 16 identified as private. Aswan, Egypt, totaled 30 unique haplotypes with 24 identified as private. One haplotype was shared by all 3 populations (H_1) and 9 haplotypes were shared by 2 populations. As with *PpSP12* and *PpSP14*, various population genetics parameters were assessed (Table 8) indicating heterogeneity among the populations. Although significant variation is present in *PpSP28*, the analyses do not indicate that positive selection is acting on this salivary protein (Table 8). Fst analysis revealed great genetic differentiation between Aswan, Egypt, and Malka, Jordan with an Fst value of 0.10913 and moderate genetic differentiation between Aswan, Egypt, and Swaimeh, Jordan with a value of 0.06936 (Table 9). There is little genetic differentiation between Malka, Jordan, and Swaimeh, Jordan, (Fst = 0.01595). The median joining network for *PpSP28* similarly does not exhibit any clear clustering of the Egypt or Jordan populations, however there are as many as 11 mutations separating connected haplotypes (Fig 9). *PpSP28* sliding window analysis of Ka/Ks demonstrates the potential for *PpSP28* to be under diversifying selection in several areas in contrast to the majority of the protein under purifying selection in all populations (Table 10). Values higher than one were detected in all 3 populations with PPJM having two sliding window regions with values over one compared to one sliding window region in PPJS and PPAW (Table 10).

**Table 8:** PpSP28 population genetics analyses for *P*. *papatasi* populations.

Population	All Data	PPAW	PPJM	PPJS
Number of Sequences	111	26	30	55
Number of Sites	554	554	554	554
- Monomorphic	495	482	485	475
- Polymorphic	95	72	69	79
Singleton variable sites	9	8	4	5
- Site positions	41, 42, 59, 94, 167, 219, 311, 392, 405	41, 59, 94, 286, 327, 389, 391, 392	8, 70, 157, 391	42, 167, 219, 311, 405
Parsimony informative sites	86	64	65	74
- Site positions	3, 6, 8, 9, 35, 40, 58, 64, 66, 67, 68, 70, 73, 79, 80, 81, 84, 93, 104, 105, 122, 129, 130, 131, 132, 142, 157, 200, 205, 206, 229, 233, 239, 250, 251, 270, 273, 286, 302, 314, 325, 327, 329, 332, 345, 349, 350, 353, 362, 365, 377, 386, 389, 391, 395, 401, 414, 416, 418, 423, 438, 444, 449, 450, 451, 459, 467, 469, 473, 476, 477, 481, 484, 486, 487, 502, 503, 510, 518, 525, 529, 530, 539, 544, 552, 553	3, 6, 8, 9, 40, 64, 67, 68, 70, 73, 79, 80, 84, 93, 105, 122, 129, 130, 131, 142, 205, 206, 229, 233, 251, 270, 273, 302, 314, 325, 329, 332, 345, 349, 350, 353, 362, 365, 386, 395, 401, 414, 416, 418, 423, 449, 450, 451, 467, 473, 476, 481, 484, 486, 502, 503, 510, 518, 525, 529, 530, 539, 552, 553	3, 6, 9, 35, 40, 58, 64, 66, 67, 68, 79, 80, 84, 93, 104, 105, 122, 129, 130, 131, 132, 200, 229, 233, 239, 250, 251, 270, 273, 286, 302, 314, 325, 329, 332, 345, 349, 350, 362, 377, 386, 395, 414, 416, 423, 438, 444, 449, 450, 459, 467, 469, 473, 476, 477, 481, 484, 486, 503, 510, 525, 529, 539, 544, 553	3, 6, 8, 9, 35, 40, 58, 64, 66, 67, 68, 70, 79, 80, 81, 84, 93, 104, 105, 122, 129, 130, 131, 132, 157, 200, 229, 233, 239, 251, 270, 273, 286, 302, 314, 325, 327, 329, 332, 345, 349, 350, 362, 377, 386, 389, 395, 414, 416, 423, 438, 444, 450, 451, 459, 467, 469, 473, 476, 477, 481, 484, 486, 487, 502, 503, 510, 518, 525, 529, 530, 539, 552, 553
Segregating sites (S)	95	72	69	79
Total number of mutations (Eta)	107	75	70	88
Total number of synonymous changes	34	27	21	26
- Site positions	35, 41, 59, 80, 104, 122, 122, 167, 200, 206, 233, 239, 251, 302, 311, 314, 332, 353, 362, 365, 377, 386, 386, 389, 392, 395, 401, 416, 423, 449, 467, 518, 525, 539	41, 59, 68, 80, 122, 206, 233, 251, 302, 332, 350, 353, 362, 365, 386, 389, 392, 395, 401, 416, 423, 449, 467, 518, 525, 530, 539	35, 80, 104, 122, 200, 233, 239, 251, 302, 329, 332, 350, 362, 377, 386, 395, 416, 423, 449, 467, 525	35, 80, 104, 122, 122, 167, 200, 233, 239, 251, 302, 311, 314, 332, 362, 377, 386, 386, 389, 395, 416, 423, 467, 518, 525, 530
Total number of replacement changes	52	42	43	44
- Site positions	3, 6, 8, 9, 40, 42, 58, 64, 70, 73, 79, 81, 84, 93, 94, 105, 132, 142, 157, 205, 219, 229, 250, 270, 273, 286, 314, 325, 345, 391, 405, 414, 418, 423, 438, 444, 450, 451, 459, 469, 473, 476, 477, 481, 484, 486, 487, 510, 539, 544, 552, 553	3, 6, 8, 9, 40, 64, 67, 70, 73, 79, 84, 93, 94, 105, 142, 205, 229, 270, 273, 286, 314, 325, 345, 349, 391, 414, 418, 423, 450, 451, 473, 476, 481, 484, 486, 502, 503, 510, 529, 539, 552, 553	3, 6, 8, 9, 40, 58, 64, 70, 79, 84, 93, 105, 132, 157, 229, 250, 270, 273, 286, 314, 325, 329, 345, 349, 391, 414, 438, 444, 450, 459, 469, 473, 476, 477, 481, 484, 486, 503, 510, 529, 539, 544, 553	3, 6, 8, 9, 40, 42, 58, 64, 70, 79, 81, 84, 93, 105, 132, 157, 219, 229, 270, 273, 286, 314, 325, 345, 405, 414, 438, 444, 450, 451, 459, 469, 473, 476, 477, 481, 484, 486, 487, 510, 529, 539, 552, 553
Number of haplotypes	122	33	28	72
Haplotype diversity (Hd)	0.9881	0.974	0.943	0.991
- Standard deviation of Hd	0.0022	0.00009	0.018	0.003
Nucleotide diversity (Pi)	0.03664	0.03798	0.03276	0.03542
- Standard deviation of Pi	0.00078	0.00114	0.00162	0.00114
Theta (per site) from S (Theta-W)	0.02869	0.02876	0.02671	0.02704
- Standard deviation of theta (no recombination)	0.00666	0.00846	0.00770	0.00703
- Standard deviation of theta (free recombination)	0.00294	0.00339	0.00322	0.00304
Theta (per site) from Pi	0.03852	0.04001	0.03426	0.03718
Average number of nucleotide differences (k)	20.299	21.04223	18.15085	19.623
Theta estimated from Eta	17.900	16.597	15.011	16.688
Fu and Li’s D test statistic	0.7669	0.97403	1.56388	1.26815
- Statistical significance	NS	NS	*	NS
Fu and Li’s F test statistic	0.71729	1.14769	1.48451	1.16499
- Statistical significance	NS	NS	NS^1^	NS
Tajima’s D	0.41277	0.93781	0.71744	0.57070
- Statistical significance	NS	NS	NS	NS
Synonymous sites Tajima’s D(Syn)	0.42640	0.61409	0.94295	0.62296
- Statistical significance	NS	NS	NS	NS
Nonsynonymous sites Tajima’s D(Nonsyn)	0.87413	1.20554	0.73109	0.77763
- Statistical significance	NS	NS	NS	NS
Silent sites Tajima’s D(Sil)	0.42640	0.61409	0.94295	0.62296
- Statistical significance	NS	NS	NS	NS
Tajima’s D (Nonsyn/Syn) ration	2.05003	1.96314	0.77532	1.24830
ω (Ka/Ks)	---	0.477	0.487	0.497

NS = *p*>0.10; NS^1^ = 0.10 > *p* > 0.05

* = *p*<0.05

**Table 9:** *PpSP28* pairwise comparisons of genetic differentiation estimates.

POP 1	POP 2	Hs	Ks	Gst	Fst	Dxy	Da
PPAW	PPJM	0.95748	19.49328	0.02012	0.10931	0.03971	0.00434
PPAW	PPJS	0.98550	20.07880	0.00712	0.06936	0.03944	0.00274
PPJM	PPJS	0.97399	19.10365	0.01061	0.01595	0.03464	0.00055

**Table 10:** PpSP28 sliding window analysis.

	Ka/Ks		
Sliding Window	PPAW	PPJM	PPJS
1–71	1.506	1.329	1.437
72–141	0.624	0.427	0.482
142–211	0.413	0.059	0.254
212–281	0.453	0.473	0.571
282–351	0.852	0.570	0.756
352–421	0.031	0.038	0.024
422–491	0.391	0.890	0.862
492–554	0.845	1.333	0.534

Ka/Ks were plotted for every 70 codons. Values greater than one suggest the potential for positive selection.

#### Secondary structure & T-cell epitope predictions

**Fig 10:**
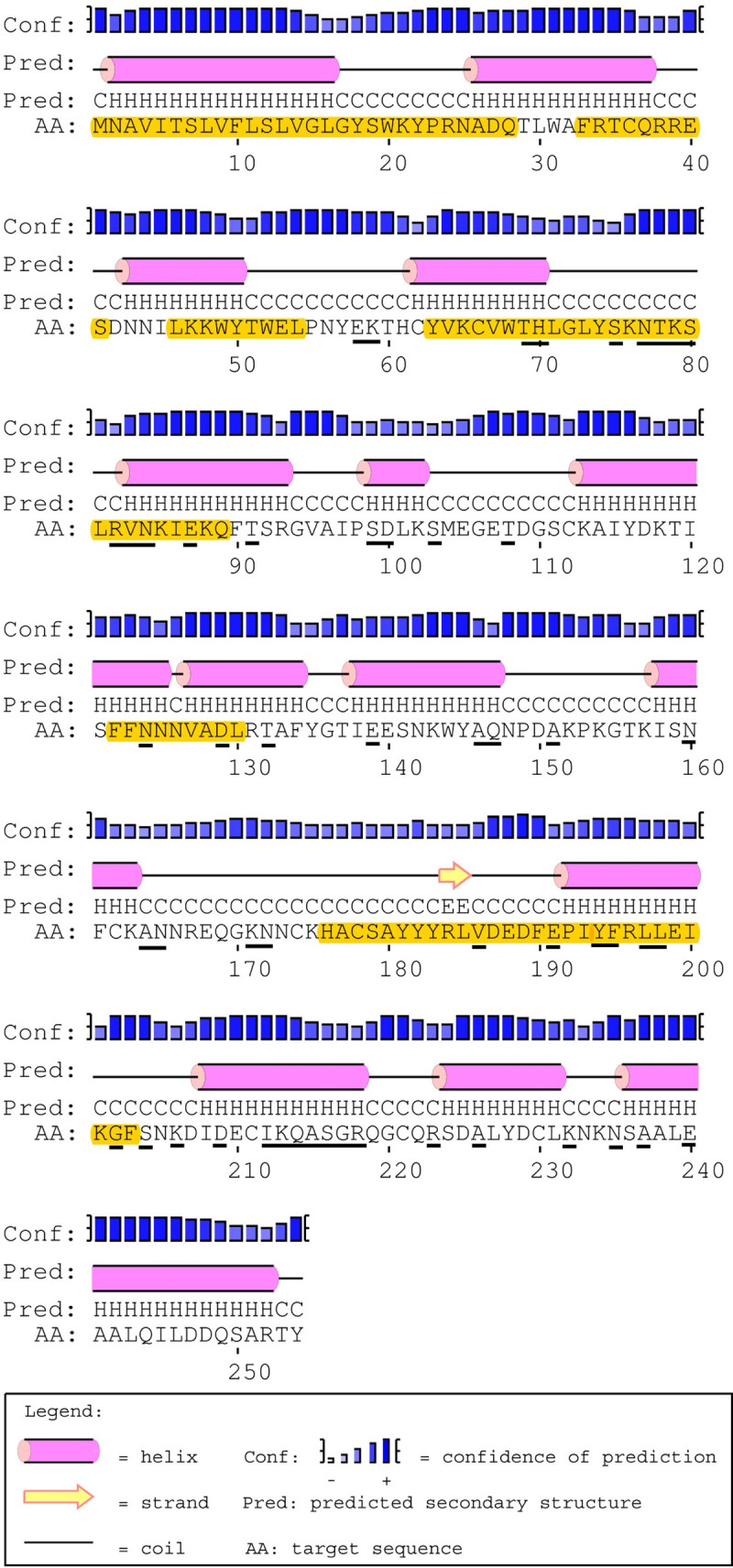
PpSp28 secondary structure, polymorphic sites, and MHC class II epitope predictions. The mature PpSP28 amino acid sequence predicted secondary structure. Yellow highlighted amino acids indicate the predicted MHC class II predicted promiscuous peptides. Individual amino acids underlined in black indicate unique polymorphic sites. Predicted secondary structure based on sequence accession #AGE83090 and #AGE83091 [51].

The predicted amino acid sequence for PpSP28 revealed 53 polymorphic sites with 30 predicted to be in α-helices whereas the other 23 polymorphic sites were found in predicted coils (Fig 10). Twenty of the polymorphic sites were found in predicted MHC II T-cell epitope binding sites. Only 1 high affinity predicted binding site between residues M1 and S19 showed no variation. IEDB identified two alleles would recognize this region including DPA1_0301/DPB1_0402 and DPA1_01/DPB1_0401. The majority of the ProPred alleles recognized some combination of residues between M1 and Q28. Another conserved region was identified between residues F33 and S41 and was recognized by DRB1_08XX, DRB1_11XX, and DRB1_13XX. The final region demonstrating conservation between residues L46 and L54 was recognized but by very few alleles. Two of the regions with the strongest affinities housed the most variation such as residues L71 to Q89, with 9 variant sites, and F195 to F203, with 4 variant sites. The high degree of variation within potential epitope binding sites and the decreased variety of alleles identified should exclude PpSP28 from further vaccine development.

### Multi-copy gene analysis for all salivary proteins

Consistent with prior vector genome assemblies, variation within and/or between loci seems to have affected the initial assembly of SP proteins in this species. In general, comparing 2 individual sand fly samples relative to the reference assembly implies that some SP proteins may exist as 2 separate loci given its coverage is significantly less than expected (p <0.0002; see Methods) using the Poisson-based coverage model of Lander and Waterman [52]. The only potential evidence of multiple copies occurred at the 3’ terminal end of the second and third region (exon) of SP42 (2-3X expectation in both samples); however, other regions of the same gene were found less than expected (p < 0.0002) (S2 Table).

## Discussion

We examined the genetic variability and potential immunogenicity of nine abundantly expressed *P*. *papatasi* salivary proteins with the overarching goal of identifying prospective targets to incorporate into an anti-leishmanial vaccine. The salivary proteins assessed included: PpSP12, PpSP14, PpSP28, PpSP29, PpSP30, PpSP32, PpSP36, PpSP42, and PpSP44. All sand flies were collected from three natural populations and were subjected to similar analyses that we previously performed for PpSP15 [14] to ascertain those salivary proteins that demonstrate similar characteristics to PpSP15 as it has been extensively studied as a vaccine target [53]. A multitude of considerations must be addressed to characterize a salivary protein as a potential vaccine candidate, including genetic variability and conservation across populations, consistent expression, and immunogenicity. We recommend that PpSP12 and PpSP14 be considered in vaccination strategies as these proteins are conserved across populations, demonstrate minimal variability, do not appear to be under selective pressure, and have the potential to activate the human immune system. PpSP28, PpSP29, PpSP32, PpSP36, PpSP42, or PpSP44 may be viable candidates for further vaccination applications but we would prioritize PpSP12 and PpSP14.

PpSP12 and PpSP14 exhibited a high degree of conservation at the nucleotide and amino acid levels (Figs 2 & 5) across all three populations studied. When our sampled sequences were aligned with previously published *P*. *papatasi* salivary protein gene sequences from Tunisia (*PpSP12* accession number JQ988874 and *PpSP14* accession number JQ988880) [51] and Israel (*PpSP12* accession number AF335485 and *PpSP14* accession number AF335486) [15], *PpSP12* and *PpSP14* demonstrated almost identical sequences with 95% and 91% identity shared, respectively. This level of conservation across multiple populations beyond those included in this study demonstrates the potential for a vaccine with broad geographic coverage. Furthermore, the population genetics indices do not indicate that PpSP12 is under selective pressure. PpSP14 might be under slight selective pressure as evidenced by Tajima’s D and Ka/Ks ratio though these values are not statistically significant (refer to Table 5 for summary information). In addition, a smaller number of nonsynonymous mutations or replacement changes are observed in PpSP12 (6) and PpSP14 (13) than in our previous PpSP15 analysis (19), further suggesting PpSP12 and PpSP14 are not under positive selection pressures [14]. Nor do the median joining networks utilizing these genes indicate any clear population structuring.

PpSP12, PpSp14, and PpSP15 belong to the family of small odorant-binding proteins (OBP) but their specific functions are unknown. *Phlebotomus* OBPs are related to the D7 protein family that includes PpSP28 and PpSP30 and may have arose from a duplication event of a D7 gene [51]. The high degree of conservancy among the OBPs demonstrated in this study mimics similar conservation of salivary proteins in geographically distant populations of *P*. *duboscqi* in Kenya and Mali [54]. The amplicon region for SP30 is 98.91% identical between the *P*. *papatasi* reference sequence and one *P*. *duboscqi* sequence, so based on the sequencing here, we cannot be absolutely sure that this sequence is from *P*. *papatasi*. However, because we did not collect any *P*. *duboscqi* and the other salivary gland genes, amplified from the same flies, exhibited more identity to *P*. *papatasi* genes than *P*. *duboscqi* genes, we are confident that these sequences are not from *P*. *dubsocqi*. *P*. *duboscqi* and *P*. *papatasi* belong to the same subgenus and are both known vectors of *Le*. *major*. The use of highly conserved salivary proteins across sand fly species to elicit a cross-protective effect would make the ideal vaccine, and cross protection against *Le*. *major* using salivary gland homogenate from *P*. *papatasi* and *P*. *duboscqi* using a murine model has been demonstrated [55]. Even though species specificity exists, cross protection may be possible across species that vector the same *Leishmania* parasites, i.e. *Le*. *major* vectored by *P*. *papatasi*, *P*. *duboscqi*, and *P*. *bergeroti*.

Gene expression is another important consideration in vaccine design as it relates to antigen dosage [56]. Salivary protein genes that are constitutively expressed are viable vaccine targets more so than those genes that change due to seasonality or other factors. We previously assessed the expression of the nine genes analyzed here from the same collections [26] and found that *PpSP12* was significantly upregulated in September for the PPJS population but no significant change occurred in the other populations. *PpSP14* did not experience a significant change in expression during the sampling season [26]. Sugar content in plants from dry habitats, like Swaimeh, Jordan, varies in comparison to plants found in irrigated areas like Aswan, Egypt, suggesting that sugar source may be a principle factor in the differential expression demonstrated by *PpSP12*. We also have demonstrated that gene expression of *PpSP12* and *PpSP14* is influenced by diet and senescence [57]. In colony-reared, 3-day old sand flies, a 3.95 and 2.18-fold change was observed in blood-fed and sugar-fed flies respectively, compared to nonfed sand flies for *PpSP12*. For *PpSP14*, there was a 3.05-fold change in blood fed females compared to nonfed female sand flies. There was similar upregulation of both *PpSP12* and *PpSP14* at day 5 and day 9 post-emergence. Though diet and senescence may influence salivary gland gene expression, environmental factors play a much larger role in gene expression regulation in wild populations [26]. Both *PpSP12* and *PpSP14* were expressed throughout seasonal trappings and when specifically tested for age or diet. Although these genes are not considered constitutively expressed like *PpSP32*, they do not experience downregulation providing further evidence of their potential to provide a high enough antigen dose to prime the immune system for protection [26, 57].

Another key aspect to vaccine development is the potential to elicit an immune response in human hosts. If certain salivary proteins are not predicted to interface with the appropriate human immune cells, then those salivary proteins should be excluded from further study. Both mature PpSP12 and PpSP14 proteins have multiple promiscuous MHC class II epitopes identified for presentation to T-cell receptors with limited variation in the potential epitope regions. Conversely, PpSP28 demonstrates high variability in predicted epitope regions decreasing the bonding likelihood with MHC class II receptors. We also identified the MHC class II alleles expected to recognize the salivary protein epitopes and investigated the predominant alleles of human populations living in Egypt and Jordan. The MHC class II alleles with strong binding affinities for PpSP12 that are also prevalent in Egyptian and Jordanian human populations include: DRB1_0301, DRB1_040X, DRB1_110X, DRB1_1301, and DRB1_150X [58–60]. The MHC class II alleles identified for PpSP14 include: DRB1_1101, DRB1_0301, DRB1_040X, DRB1_11XX, DRB1_13XX, DRB1_15XX, and to a lesser extent DRB1_070X [58–60]. The six remaining salivary proteins were predicted to bind to MHC class II alleles with varying affinity. PpSP36, PpSp42, and PpSP44 demonstrated greater binding affinities to multiple regions for each predicted protein structure but were not predicted to bind with the most prevalent alleles in the human populations from Egypt and Jordan (Supl. data). PpSP29, PpSP30, and PpSP32, displayed fewer predicted binding regions with lower affinities for those regions (Supl. data).

Of critical importance is whether these salivary proteins are recognized by human plasma. Although PpSP12 and PpSP14 are smaller in size than the other salivary proteins analyzed, they are less variable overall as there is less opportunity for mutations to occur. Even though larger proteins might be more immunogenic, our data, supported by previous studies, indicate that PpSP12 and PpSP14 will be recognized by alleles circulating in the study areas [16, 58–60]. Both PpSP12 and PpSP14 are recognized by the immune system but antibody specificity differs among the human populations tested [16]. Our previous assessment of human responses included Egyptian and Jordanian residents (MENA donors) from the same study sites analyzed here and U.S. military personnel deployed overseas [16]; MENA donors displayed antibody responses to PpSP12, PpSP26, PpSP30, PpSP38, and PpSP44 but not PpSP14, whereas U.S. military displayed specificity to PpSP14 and PpSP38 but not PpSP12 [16]. In an independent study, it was shown that plasma antibody specificity of 200 Tunisian children ages 6 to 12 years old reacted to PpSP12, PpSP15, PpSP21, PpSP28, PpSP30, PpSP36, and PpSP44, but not to PpSP14 [61], emphasizing the impact of prolonged exposure to sand fly bites versus naïve individuals traveling to sand fly endemic areas [16, 61]. Interestingly, PpSP12 and PpSP14 were also shown immunoreactive in unexposed control donors and that the circulating antibodies against these specific salivary proteins could be the result of exposure to other hematophagous arthropod species [16].

Specific antibody response also factors into the polarization of the immune response to a Th1-mediated or Th2-mediated response. The polarization to Th1 or Th2 responses result in protection against CL or a disease exacerbation effect, respectively [10, 15]. In our previous work, total *P*. *papatasi* salivary gland homogenate elicited IgG4 specificity as the dominant isotype and subclass circulating in human donors and positively correlated with IgE concentrations [16]. IgG4 and IgE are hallmarks of a Th2 and allergic hypersensitivity response [62]. Another study demonstrated that whole salivary gland homogenate upregulates interleukin 4 (IL-4) while inhibiting interleukin 12 (IL-12) and IFN-γ skewing to a Th2 response in the murine model [63]. Th1/Th2 polarization is also dependent on no exposure or pre-exposure to sand fly bites (as reviewed in [64]). The antibody response to individual salivary protein antigens was characterized [61]. PpSP12 was recognized predominately by IgG1 and IgG2 and not IgG4 nor IgE indicating its potential to polarize to a protective Th1 response. PpSP14 was not characterized as it did not demonstrate antibody specificity, but in another study produced a humoral response [5, 61].

Taken together, our results and those of others demonstrate the potential of PpSP12 and PpSp14 as vaccine targets. Further testing needs to be conducted to more specifically determine the Th1/Th2 response of PpSP12 and PpSP14 as well as determine if these proteins would confer protection in individuals living in endemic regions as well as naïve populations who may work or travel to endemic areas.

## Supporting information

S1 Table *Phlebotomus papatasi* salivary protein primers and GenBank accession numbers. *** = Amplicon is under 200 base pairs; not assigned an accession number; sequences available upon request.
(DOCX)

S2 Table *Phlebotomus papatasi* salivary protein gene multi-copy assessment. (XLS)

S3 Table PpSP29 population genetics analyses for *P*. *papatasi* populations NS = *p*>0.10; NS^1^ = 0.10 > *p* > 0.05; * = *p*<0.05. (DOCX)

S4 Table *PpSP29* pairwise comparisons of genetic differentiation estimates. (DOCX)

S5 Table PpSP29 sliding window analysis. Ka/Ks were plotted for every 70 codons. Values greater than one suggest the potential for positive selection. ----indicates a lack of polymorphic data in the window to calculate a Ka/Ks value.
(DOCX)

S6 Table PpSP30 population genetics analyses for *P*. *papatasi* populations NS = *p*>0.10; NS^1^ = 0.10 > *p* > 0.05; * = *p*<0.05. (DOCX)

S7 Table *PpSP30* pairwise comparisons of genetic differentiation estimates. (DOCX)

S8 Table PpSP30 sliding window analysis. Ka/Ks were plotted for every 70 codons. Values greater than one suggest the potential for positive selection. ----indicates a lack of polymorphic data in the window to calculate a Ka/Ks value.
(DOCX)

S9 Table PpSP32 population genetics analyses for *P*. *papatasi* populations NS = *p*>0.10; NS^1^ = 0.10 > *p* > 0.05; * = *p*<0.05. (DOCX)

S10 Table *PpSP32* pairwise comparisons of genetic differentiation estimates. (DOCX)

S11 Table PpSP32 sliding window analysis. Ka/Ks were plotted for every 70 codons. Values greater than one suggest the potential for positive selection. ----indicates a lack of polymorphic data in the window to calculate a Ka/Ks value.
(DOCX)

S12 Table PpSP36 population genetics analyses for *P*. *papatasi* populations NS = *p*>0.10; NS^1^ = 0.10 > *p* > 0.05; * = *p*<0.05. (DOCX)

S13 Table *PpSP36* pairwise comparisons of genetic differentiation estimates. (DOCX)

S14 Table PpSP36 sliding window analysis. Ka/Ks were plotted for every 70 codons. Values greater than one suggest the potential for positive selection. ----indicates a lack of polymorphic data in the window to calculate a Ka/Ks value.
(DOCX)

S15 Table PpSP42 population genetics analyses for *P*. *papatasi* populations NS = *p*>0.10; NS^1^ = 0.10 > *p* > 0.05; * = *p*<0.05. (DOCX)

S16 Table *PpSP42* pairwise comparisons of genetic differentiation estimates. (DOCX)

S17 Table PpSP42 sliding window analysis. Ka/Ks were plotted for every 70 codons. Values greater than one suggest the potential for positive selection. ----indicates a lack of polymorphic data in the window to calculate a Ka/Ks value.
(DOCX)

S18 Table PpSP44 population genetics analyses for *P*. *papatasi* populations NS = *p*>0.10; NS^1^ = 0.10 > *p* > 0.05; * = *p*<0.05. (DOCX)

S19 Table *PpSP44* pairwise comparisons of genetic differentiation estimates. (DOCX)

S20 Table PpSP44 sliding window analysis. Ka/Ks were plotted for every 70 codons. Values greater than one suggest the potential for positive selection. ----indicates a lack of polymorphic data in the window to calculate a Ka/Ks value.
(DOCX)

S21 Table Summary Tajima’s D and Ka/Ks analysis for all *P*. *papatasi* salivary proteins studied. NS = *p*>0.10; NS^1^ = 0.10 > *p* > 0.05; * = *p*<0.05.
(DOCX)

S1 Fig PpSP29 nucleotide and amino acid variation. (A) Weblogo illustrating the relative frequencies of nucleotide polymorphisms in wild caught *P*. *papatasi* populations from PPAW, PPJM, and PPJS. (B) Weblogo illustrating the relative frequencies of amino acid polymorphisms in wild caught *P*. *papatasi* populations from PPAW, PPJM, and PPJS.
(PPTX)

S2 Fig Median-joining network for PpSP29 *P*. *papatasi* haplotypes. Circle size and circle color indicates frequency and geographical location of haplotypes, respectively. Haplotype numbers are indicated next to the corresponding circle H_XX. Red numbers between haplotypes indicate number of mutations between haplotypes.
(PPTX)

S3 Fig PpSp29 secondary structure, polymorphic sites, and MHC class II epitope predictions. The mature PpSP29 amino acid sequence predicted secondary structure. Yellow highlighted amino acids indicate the predicted MHC class II predicted promiscuous peptides. Individual amino acids underlined in black indicate unique polymorphic sites. Predicted secondary structure based on sequence accession #AGE83096 [51].
(PPTX)

S4 Fig PpSP30 nucleotide and amino acid variation. (A) Weblogo illustrating the relative frequencies of nucleotide polymorphisms in wild caught *P*. *papatasi* populations from PPAW, PPJM, and PPJS. (B) Weblogo illustrating the relative frequencies of amino acid polymorphisms in wild caught *P*. *papatasi* populations from PPAW, PPJM, and PPJS.
(PPTX)

S5 Fig Median-joining network for PpSP30 *P*. *papatasi* haplotypes. Circle size and circle color indicates frequency and geographical location of haplotypes, respectively. Haplotype numbers are indicated next to the corresponding circle H_XX. Red numbers between haplotypes indicate number of mutations between haplotypes.
(PPTX)

S6 Fig PpSp30 secondary structure, polymorphic sites, and MHC class II epitope predictions. The mature PpSP30 amino acid sequence predicted secondary structure. Yellow highlighted amino acids indicate the predicted MHC class II predicted promiscuous peptides. Individual amino acids underlined in black indicate unique polymorphic sites. Predicted secondary structure based on sequence accession #AGE83093 [51].
(PPTX)

S7 Fig PpSP32 nucleotide and amino acid variation. (A) Weblogo illustrating the relative frequencies of nucleotide polymorphisms in wild caught *P*. *papatasi* populations from PPAW, PPJM, and PPJS. (B) Weblogo illustrating the relative frequencies of amino acid polymorphisms in wild caught *P*. *papatasi* populations from PPAW, PPJM, and PPJS.
(PPTX)

S8 Fig Median-joining network for PpSP32 *P*. *papatasi* haplotypes. Circle size and circle color indicates frequency and geographical location of haplotypes, respectively. Haplotype numbers are indicated next to the corresponding circle H_XX. Red numbers between haplotypes indicate number of mutations between haplotypes.
(PPTX)

S9 Fig PpSp32 secondary structure, polymorphic sites, and MHC class II epitope predictions. The mature PpSP32 amino acid sequence predicted secondary structure. Yellow highlighted amino acids indicate the predicted MHC class II predicted promiscuous peptides. Individual amino acids underlined in black indicate unique polymorphic sites. Predicted secondary structure based on sequence accession #AGE83097 [51].
(PPTX)

S10 Fig PpSP36 nucleotide and amino acid variation. (A) Weblogo illustrating the relative frequencies of nucleotide polymorphisms in wild caught *P*. *papatasi* populations from PPAW, PPJM, and PPJS. (B) Weblogo illustrating the relative frequencies of amino acid polymorphisms in wild caught *P*. *papatasi* populations from PPAW, PPJM, and PPJS.
(PPTX)

S11 Fig Median-joining network for PpSP36 *P*. *papatasi* haplotypes. Circle size and circle color indicates frequency and geographical location of haplotypes, respectively. Haplotype numbers are indicated next to the corresponding circle H_XX. Red numbers between haplotypes indicate number of mutations between haplotypes.
(PPTX)

S12 Fig PpSp36 secondary structure, polymorphic sites, and MHC class II epitope predictions. The mature PpSP36 amino acid sequence predicted secondary structure. Yellow highlighted amino acids indicate the predicted MHC class II predicted promiscuous peptides. Individual amino acids underlined in black indicate unique polymorphic sites. Predicted secondary structure based on sequence accession #AGE83101 [51].
(PPTX)

S13 Fig PpSP42 nucleotide and amino acid variation. (A) Weblogo illustrating the relative frequencies of nucleotide polymorphisms in wild caught *P*. *papatasi* populations from PPAW, PPJM, and PPJS. (B) Weblogo illustrating the relative frequencies of amino acid polymorphisms in wild caught *P*. *papatasi* populations from PPAW, PPJM, and PPJS.
(PPTX)

S14 Fig Median-joining network for PpSP42 *P*. *papatasi* haplotypes. Circle size and circle color indicates frequency and geographical location of haplotypes, respectively. Haplotype numbers are indicated next to the corresponding circle H_XX. Red numbers between haplotypes indicate number of mutations between haplotypes.
(PPTX)

S15 Fig PpSp42 secondary structure, polymorphic sites, and MHC class II epitope predictions. The mature PpSP42 amino acid sequence predicted secondary structure. Yellow highlighted amino acids indicate the predicted MHC class II predicted promiscuous peptides. Individual amino acids underlined in black indicate unique polymorphic sites. Predicted secondary structure based on sequence accession #AGE83094 [51].
(PPTX)

S16 Fig PpSP44 nucleotide and amino acid variation. (A) Weblogo illustrating the relative frequencies of nucleotide polymorphisms in wild caught *P*. *papatasi* populations from PPAW, PPJM, and PPJS. (B) Weblogo illustrating the relative frequencies of amino acid polymorphisms in wild caught *P*. *papatasi* populations from PPAW, PPJM, and PPJS.
(PPTX)

S17 Fig Median-joining network for PpSP44 *P*. *papatasi* haplotypes. Circle size and circle color indicates frequency and geographical location of haplotypes, respectively. Haplotype numbers are indicated next to the corresponding circle H_XX. Red numbers between haplotypes indicate number of mutations between haplotypes.
(PPTX)

S18 Fig PpSp44 secondary structure, polymorphic sites, and MHC class II epitope predictions. The mature PpSP44 amino acid sequence predicted secondary structure. Yellow highlighted amino acids indicate the predicted MHC class II predicted promiscuous peptides. Individual amino acids underlined in black indicate unique polymorphic sites. Predicted secondary structure based on sequence accession #AGE83095 [51].
(PPTX)
