# Supplementary material for: Phlebotomus papatasi sand fly predicted salivary protein diversity and immune response potential based on in silico prediction in Egypt and Jordan populations
Source: PLoS Negl Trop Dis. 2020 Jul 13;14(7):e0007489. doi: 10.1371/journal.pntd.0007489 (PMC7377520; doi:10.1371/journal.pntd.0007489)
Supplement: S1 Table — *** = Amplicon is under 200 base pairs; not assigned an accession number; sequences available upon request. (DOCX) [file pntd.0007489.s001.docx]

**S1 Table. *Phlebotomus papatasi* salivary protein primers and GenBank accession numbers.**

| Salivary Protein | Forward Primer | Reverse Primer | GenBank Accession Numbers |
| --- | --- | --- | --- |
| PpSP12 | CTCAATCCTTCTAGGAAATG | TTAGTTTAAATTGATTTTTTTGTC | MK426699 - MK426727 |
| PpSP14 | TTTGAACATCCCGAAGCC | CTAGTTCTTTCTAGTTAC | MK439961 – MK43998 |
| PpSP28 | TGGAAGTACCCTAGGAATGCC | CTAGTACGTTCTTGCGGATTGGTC | MK487490 - MK487611 |
| PpSP29 | GTGACAGAGAACTGTGC | TTATTGGCCTCCTCTTCTATG | MK449450 - MK449561 |
| PpSP30 | TGGCGATTTCCTAGGAATGG | TTAGTATTTCCAAGATTC | *** |
| PpSP32 | GCAAGCACAATTCCCATTC | TCAAGCCTTGAAAGTTTTG | MK449562 - MK449721 |
| PpSP36 | GCTCCAAGAAGTGGAACAATC | TTATTGACTGCGTTTCAAAAATCC | MK449722 - MK449831 |
| PpSP42 | GATGATGTTGGAAGGGCTTATG | TTACCCTTGACACTTTTCTCC | MK449832 - MK449964 |
| PpSP44 | GACGATGTTGAAAGATTTTAC | TTATTTAGGTTTTCTCACTTC | MK449965 - MK450029 |

***=Amplicon is under 200 base pairs; not assigned an accession number; sequences available upon request.
