# Supplementary material for: Phlebotomus papatasi sand fly predicted salivary protein diversity and immune response potential based on in silico prediction in Egypt and Jordan populations
Source: PLoS Negl Trop Dis. 2020 Jul 13;14(7):e0007489. doi: 10.1371/journal.pntd.0007489 (PMC7377520; doi:10.1371/journal.pntd.0007489)
Supplement: S3 Table — (DOCX) [file pntd.0007489.s003.docx]

**S3 Table**. **PpSP29 population genetics analyses for *P. papatasi* populations.**

| Population | All Data | PPAW | PPJM | PPJS |
| --- | --- | --- | --- | --- |
| Number of Sequences | 126 | 46 | 38 | 42 |
| Number of Sites | 651 | 651 | 651 | 651 |
| - Monomorphic | 573 | 594 | 601 | 603 |
| - Polymorphic | 78 | 57 | 50 | 48 |
| Singleton variable sites | 16 | 7 | 5 | 13 |
| - Site positions | 5, 90, 108, 113, 301, 309, 358, 444, 485, 511, 549, 558, 563, 573, 579, 592 | 15, 108, 113, 358, 444, 485, 558 | 301, 468, 549, 579, 592 | 42, 90, 195, 279, 309, 336, 339, 383, 384, 405, 511, 563, 573 |
| Parsimony informative sites | 62 | 50 | 45 | 35 |
| - Site positions | 34, 35, 36, 41, 42, 45, 63, 64, 69, 81, 104, 105, 106, 109, 111, 120, 172, 180, 195, 223, 228, 243, 249, 270, 273, 279, 281, 294, 298, 303, 333, 336, 339, 345, 351, 375, 383, 384, 393, 405, 411, 414, 423, 429, 438, 447, 450, 453, 454, 459, 468, 486, 534, 537, 540, 555, 572, 594, 609, 615, 627, 645 | 35, 36, 41, 42, 45, 81, 104, 105, 106, 109, 111, 120, 172, 180, 195, 223, 228, 243, 249, 270, 273, 279, 281, 294, 298, 345, 351, 375, 383, 384, 393, 405, 411, 423, 438, 447, 450, 453, 454, 468, 486, 534, 537, 540, 572, 594, 609, 615, 627, 645 | 34, 35, 36, 41, 45, 63, 64, 69, 109, 120, 172, 180, 223, 249, 270, 273, 281, 294, 298, 303, 333, 336, 339, 345, 375, 383, 384, 393, 405, 411, 414, 423, 429, 447, 450, 453, 454, 486, 534, 537, 540, 555, 572, 609, 615 | 35, 36, 41, 45, 109, 120, 172, 180, 223, 270, 273, 281, 298, 303, 333, 345, 375, 393, 411, 423, 429, 447, 450, 453, 454, 459, 468, 486, 534, 537, 540, 555, 572, 609, 645 |
| Segregating sites (S) | 78 | 57 | 50 | 48 |
| Total number of mutations (Eta) | 83 | 60 | 52 | 51 |
| Total number of synonymous changes | 60 | 44 | 39 | 39 |
| - Site positions | 15, 42, 45, 63, 64, 69, 81, 90, 105, 108, 111, 120, 172, 180, 195, 228, 243, 249, 270, 273, 279, 294, 303, 309, 333, 336, 339, 345, 351, 375, 393, 393, 405, 411, 414, 423, 429, 438, 444, 447, 447, 450, 453, 453, 459, 468, 486, 534, 537, 540, 549, 555, 558, 573, 579, 594, 609, 615, 627, 645 | 15, 36, 42, 45, 81, 105, 108, 111, 120, 172, 180, 195, 228, 243, 249, 270, 273, 279, 294, 345, 351, 375, 393, 405, 411, 423, 438, 444, 447, 447, 450, 453, 453, 468, 486, 534, 537, 540, 558, 594, 609, 615, 627, 645 | 45, 63, 64, 69, 120, 172, 180, 249, 270, 273, 294, 303, 333, 336, 339, 345, 375, 384, 393, 393, 405, 411, 414, 423, 429, 447, 447, 450, 453, 468, 486, 534, 537, 540, 549, 555, 579, 609, 615 | 36, 42, 45, 90, 120, 172, 180, 195, 270, 273, 279, 303, 309, 333, 336, 339, 345, 375, 384, 393, 393, 405, 411, 423, 429, 447, 450, 453, 453, 459, 468, 486, 534, 537, 540, 555, 573, 609, 645 |
| Total number of replacement changes | 17 | 13 | 10 | 12 |
| - Site positions | 41, 104, 106, 109, 113, 223, 281, 281, 298, 301, 358, 454, 485, 511, 563, 572, 592 | 35, 41, 104, 106, 109, 113, 223, 281, 298, 358, 454, 485, 572 | 41, 109, 223, 281, 298, 301, 383, 454, 572, 592 | 35, 41, 109, 223, 281, 281, 298, 383, 454, 511, 563, 572 |
| Number of haplotypes | 112 | 47 | 30 | 39 |
| Haplotype diversity (Hd) | 0.9480 | 0.945 | 0.859 | 0.906 |
| - Standard deviation of Hd | 0.0100 | 0.014 | 0.038 | 0.026 |
| Nucleotide diversity (Pi) | 0.01598 | 0.01812 | 0.01382 | 0.01339 |
| - Standard deviation of Pi | 0.00041 | 0.00051 | 0.00089 | 0.00062 |
| Theta (per site) from S (Theta-W) | 0.01963 | 0.01719 | 0.01567 | 0.01474 |
| - Standard deviation of theta (no recombination) | 0.00458 | 0.00473 | 0.00450 | 0.00419 |
| - Standard deviation of theta (free recombination) | 0.00222 | 0.00228 | 0.00222 | 0.00213 |
| Theta (per site) from Pi | 0.01633 | 0.01857 | 0.01408 | 0.01363 |
| Average number of nucleotide differences (k) | 10.403 | 11.797 | 8.998 | 8.716 |
| Theta estimated from Eta | 13.596 | 11.780 | 10.609 | 10.196 |
| Fu and Li’s D test statistic | -0.85227 | 0.54253 | 1.13071 | -1.17588 |
| - Statistical significance | NS | NS | NS | NS |
| Fu and Li’s F test statistic | -0.94063 | 0.38862 | 0.59010 | -1.07371 |
| - Statistical significance | NS | NS | NS | NS |
| Tajima’s D | -0.70794 | 0.00484 | -0.49866 | -0.47054 |
| - Statistical significance | NS | NS | NS | NS |
| Synonymous sites Tajima’s D(Syn) | -0.58472 | 0.20515 | -0.50512 | -0.46706 |
| - Statistical significance | NS | NS | NS | NS |
| Nonsynonymous sites Tajima’s D(Nonsyn) | -0.89172 | -0.47007 | -0.24454 | -0.37957 |
| - Statistical significance | NS | NS | NS | NS |
| Silent sites Tajima’s D(Sil) | -0.58472 | 0.20515 | -0.50512 | -0.46706 |
| - Statistical significance | NS | NS | NS | NS |
| Tajima’s D (Nonsyn/Syn) ration | 1.52504 | -2.29133 | 0.48413 | 0.81270 |
| ω (Ka/Ks) | --- | 0.066 | 0.082 | 0.080 |

NS=*p*>0.10; NS^1^=0.10 > *p* > 0.05; *=*p*<0.05
