# Supplementary material for: Phlebotomus papatasi sand fly predicted salivary protein diversity and immune response potential based on in silico prediction in Egypt and Jordan populations
Source: PLoS Negl Trop Dis. 2020 Jul 13;14(7):e0007489. doi: 10.1371/journal.pntd.0007489 (PMC7377520; doi:10.1371/journal.pntd.0007489)
Supplement: S4 Table — (DOCX) [file pntd.0007489.s004.docx]

**S4 Table. *PpSP29* pairwise comparisons of genetic differentiation estimates.**

| POP 1 | POP 2 | Hs | Ks | Gst | Fst | Dxy | Da |
| --- | --- | --- | --- | --- | --- | --- | --- |
| PPAW | PPJM | 0.90649 | 10.53100 | 0.05019 | 0.07425 | 0.01725 | 0.00128 |
| PPAW | PPJS | 0.92652 | 10.32676 | 0.03841 | 0.08877 | 0.01729 | 0.00153 |
| PPJM | PPJS | 0.88380 | 8.85022 | 0.00647 | 0.02630 | 0.01397 | 0.00037 |
