# Supplementary material for: Phlebotomus papatasi sand fly predicted salivary protein diversity and immune response potential based on in silico prediction in Egypt and Jordan populations
Source: PLoS Negl Trop Dis. 2020 Jul 13;14(7):e0007489. doi: 10.1371/journal.pntd.0007489 (PMC7377520; doi:10.1371/journal.pntd.0007489)
Supplement: S5 Table — Ka/Ks were plotted for every 70 codons. Values greater than one suggest the potential for positive selection. ----indicates a lack of polymorphic data in the window to calculate a Ka/Ks value. (DOCX) [file pntd.0007489.s005.docx]

**S5 Table. PpSP29 sliding window analysis.**

|  | Ka/Ks | | |
| --- | --- | --- | --- |
| Sliding Window | PPAW | PPJM | PPJS |
| 1-70 | 0.136 | 0.211 | 0.173 |
| 71-140 | 0.123 | 0.187 | 0.389 |
| 141-210 | 0.000 | 0.000 | 0.000 |
| 211-280 | 0.043 | 0.130 | 0.193 |
| 281-350 | 0.334 | 0.262 | 0.428 |
| 351-420 | 0.052 | 0.025 | 0.007 |
| 421-490 | 0.036 | 0.012 | 0.005 |
| 491-560 | 0.000 | 0.000 | 0.008 |
| 561-630 | 0.026 | 0.097 | 0.048 |
| 631-651 | 0.000 | 0.000 | 0.000 |

Ka/Ks were plotted for every 70 codons. Values greater than one suggest the potential for positive selection. ---- indicates a lack of polymorphic data in the window to calculate a Ka/Ks value.
