# Supplementary material for: Phlebotomus papatasi sand fly predicted salivary protein diversity and immune response potential based on in silico prediction in Egypt and Jordan populations
Source: PLoS Negl Trop Dis. 2020 Jul 13;14(7):e0007489. doi: 10.1371/journal.pntd.0007489 (PMC7377520; doi:10.1371/journal.pntd.0007489)
Supplement: S6 Table — (DOCX) [file pntd.0007489.s006.docx]

**S6 Table**. **PpSP30 population genetics analyses for *P. papatasi* populations**

| Population | All Data | PPAW | PPJM | PPJS |
| --- | --- | --- | --- | --- |
| Number of Sequences | 70 | 20 | 28 | 22 |
| Number of Sites | 183 | 183 | 183 | 183 |
| - Monomorphic | 162 | 171 | 169 | 174 |
| - Polymorphic | 21 | 12 | 14 | 9 |
| Singleton variable sites | 0 | 0 | 1 | 0 |
| - Site positions | -- | --- | 77 | --- |
| Parsimony informative sites | 21 | 12 | 13 | 9 |
| - Site positions | 6 25 28 37 47 61 77 85 87 109 116 122 133 148 149 151 153 154 175 178 181 | 6 25 37 47 61 85 109 116 133 175 178 181 | 25 28 37 47 61 87 109 122 148 149 153 154 175 | 25 28 37 47 77 109 122 151 154 |
| Segregating sites (S) | 21 | 12 | 14 | 9 |
| Total number of mutations (Eta) | 24 | 13 | 14 | 11 |
| Total number of synonymous changes | 12 | 9 | 6 | 5 |
| - Site positions | 25 28 37 85 109 116 133 148 151 175 178 181 | 25 37 85 109 116 133 175 178 181 | 25 28 37 109 148 175 | 25 28 37 109 151 |
| Total number of replacement changes | 12 | 4 | 8 | 6 |
| - Site positions | 6 37 47 61 77 87 109 109 122 149 153 154 | 6 47 61 109 | 47 61 77 87 122 149 153 154 | 37 47 77 109 122 154 |
| Number of haplotypes | 35 | 15 | 21 | 12 |
| Haplotype diversity (Hd) | 0.938 | 0.923 | 0.914 | 0.904 |
| - Standard deviation of Hd | 0.008 | 0.020 | 0.023 | 0.020 |
| Nucleotide diversity (Pi) | 0.01359 | 0.01275 | 0.01303 | 0.01166 |
| - Standard deviation of Pi | 0.00065 | 0.00119 | 0.00117 | 0.00100 |
| Theta (per site) from S (Theta-W) | 0.02081 | 0.01542 | 0.01665 | 0.01131 |
| - Standard deviation of theta (no recombination) | 0.00646 | 0.00614 | 0.00618 | 0.00481 |
| - Standard deviation of theta (free recombination) | 0.00454 | 0.00445 | 0.00445 | 0.00377 |
| Theta (per site) from Pi | 0.01384 | 0.01297 | 0.01326 | 0.01185 |
| Average number of nucleotide differences (k) | 2.486 | 2.333 | 2.385 | 2.134 |
| Theta estimated from Eta | 4.352 | 3.056 | 3.048 | 2.529 |
| Fu and Li’s D test statistic | 0.58468 | 1.02573 | 1.04773 | 0.32710 |
| - Statistical significance | NS | NS | NS | NS |
| Fu and Li’s F test statistic | -0.15252 | 0.53185 | 0.55470 | 0.08128 |
| - Statistical significance | NS | NS | NS | NS |
| Tajima’s D | -1.22339 | -0.73802 | -0.64753 | -0.46491 |
| - Statistical significance | NS | NS | NS | NS |
| Synonymous sites Tajima’s D(Syn) | -0.61160 | -0.51243 | 0.11820 | 0.50544 |
| - Statistical significance | NS | NS | NS | NS |
| Nonsynonymous sites Tajima’s D(Nonsyn) | -0.44881 | -0.31085 | -1.11170 | 0.77367 |
| - Statistical significance | NS | NS | NS | NS |
| Silent sites Tajima’s D(Sil) | -0.61160 | -0.51243 | 0.11820 | 0.50544 |
| - Statistical significance | NS | NS | NS | NS |
| Tajima’s D (Nonsyn/Syn) ratio | 0.73383 | 0.60663 | -9.40516 | 1.53070 |
| ω (Ka/Ks) | ---- | 0.095 | 0.190 | 0.166 |

NS=*p*>0.10; NS^1^=0.10 > *p* > 0.05; *=*p*<0.05
