# Supplementary material for: Phlebotomus papatasi sand fly predicted salivary protein diversity and immune response potential based on in silico prediction in Egypt and Jordan populations
Source: PLoS Negl Trop Dis. 2020 Jul 13;14(7):e0007489. doi: 10.1371/journal.pntd.0007489 (PMC7377520; doi:10.1371/journal.pntd.0007489)
Supplement: S7 Table — (DOCX) [file pntd.0007489.s007.docx]

**S7 Table. *PpSP30* pairwise comparisons of genetic differentiation estimates.**

| POP 1 | POP 2 | Hs | Ks | Gst | Fst | Dxy | Da |
| --- | --- | --- | --- | --- | --- | --- | --- |
| PPAW | PPJM | 0.91754 | 2.36351 | 0.02629 | 0.15229 | 0.1521 | 0.00232 |
| PPAW | PPJS | 0.91296 | 2.22905 | 0.03315 | 0.18670 | 0.01501 | 0.00280 |
| PPJM | PPJS | 0.90934 | 2.27471 | 0.00451 | 0.00752 | 0.01244 | 0.00009 |
