# Supplementary material for: Phlebotomus papatasi sand fly predicted salivary protein diversity and immune response potential based on in silico prediction in Egypt and Jordan populations
Source: PLoS Negl Trop Dis. 2020 Jul 13;14(7):e0007489. doi: 10.1371/journal.pntd.0007489 (PMC7377520; doi:10.1371/journal.pntd.0007489)
Supplement: S8 Table — Ka/Ks were plotted for every 70 codons. Values greater than one suggest the potential for positive selection. ----indicates a lack of polymorphic data in the window to calculate a Ka/Ks value. (DOCX) [file pntd.0007489.s008.docx]

**S8 Table. PpSP30 sliding window analysis.**

|  | Ka/Ks | | |
| --- | --- | --- | --- |
| Sliding Window | PPAW | PPJM | PPJS |
| 1-70 | 0.299 | 0.143 | 0.118 |
| 71-140 | 0.022 | 0.162 | 0.230 |
| 141-183 | 0.000 | 0.451 | 0.229 |

Ka/Ks were plotted for every 70 codons. Values greater than one suggest the potential for positive selection. ---- indicates a lack of polymorphic data in the window to calculate a Ka/Ks value.
