# Supplementary material for: Phlebotomus papatasi sand fly predicted salivary protein diversity and immune response potential based on in silico prediction in Egypt and Jordan populations
Source: PLoS Negl Trop Dis. 2020 Jul 13;14(7):e0007489. doi: 10.1371/journal.pntd.0007489 (PMC7377520; doi:10.1371/journal.pntd.0007489)
Supplement: S9 Table — (DOCX) [file pntd.0007489.s009.docx]

**S9 Table**. **PpSP32 population genetics analyses for *P. papatasi* populations.**

| Population | All Data | PPAW | PPJM | PPJS |
| --- | --- | --- | --- | --- |
| Number of Sequences | 130 | 42 | 45 | 43 |
| Number of Sites | 568 | 568 | 568 | 568 |
| - Monomorphic | 523 | 533 | 537 | 543 |
| - Polymorphic | 45 | 35 | 31 | 25 |
| Singleton variable sites | 11 | 7 | 5 | 2 |
| - Site positions | 14, 74, 119, 120, 129, 236, 239, 240, 404, 421, 450 | 14, 74, 120, 129, 246, 404, 421 | 119, 236, 239, 241, 450 | 240, 272 |
| Parsimony informative sites | 34 | 28 | 26 | 23 |
| - Site positions | 46, 67, 72, 75, 79, 80, 85, 97, 113, 128, 157, 216, 230, 235, 241, 246, 272, 294, 296, 355, 402, 428, 430, 436, 446, 454, 479, 485, 510, 533, 535, 539, 540, 546 | 46, 67, 72, 75, 79, 80, 85, 97, 113, 128, 157, 216, 230, 241, 294, 296, 355, 430, 436, 446, 454, 479, 485, 510, 535, 539, 540, 546 | 46, 75, 79, 80, 97, 113, 128, 157, 216, 235, 246, 272, 294, 296, 355, 428, 430, 436, 446, 454, 479, 485, 533, 535, 540, 546 | 46, 75, 79, 80, 85, 97, 113, 128, 157, 294, 296, 355, 402, 436, 446, 454, 479, 485, 510, 535, 539, 540, 546 |
| Segregating sites (S) | 45 | 35 | 31 | 25 |
| Total number of mutations (Eta) | 46 | 36 | 31 | 25 |
| Total number of synonymous changes | 11 | 11 | 9 | 7 |
| - Site positions | 46, 67, 85, 97, 157, 235, 355, 421, 430, 436, 454 | 46, 67, 85, 97, 157, 241, 355, 421, 430, 436, 454 | 46, 97, 157, 235, 241, 355, 430, 436, 454 | 46, 85, 97, 157, 355, 436, 454 |
| Total number of replacement changes | 29 | 22 | 22 | 18 |
| - Site positions | 14, 72, 74, 75, 79, 80, 113, 119, 120, 128, 129, 216, 230, 236, 246, 272, 294, 296, 402, 404, 428, 446, 450, 479, 485, 510, 533, 535, 546 | 14, 72, 74, 75, 79, 80, 113, 120, 128, 129, 216, 230, 246, 294, 296, 404, 446, 479, 485, 510, 535, 546 | 75, 79, 80, 113, 119, 128, 216, 236, 239, 246, 272, 294, 296, 428, 446, 450, 479, 485, 533, 535, 540, 546 | 75, 79, 80, 113, 128, 240, 272, 294, 296, 402, 446, 479, 485, 510, 535, 539, 540, 546 |
| Number of haplotypes | 160 | 59 | 63 | 58 |
| Haplotype diversity (Hd) | 0.9870 | 0.981 | 0.985 | 0.983 |
| - Standard deviation of Hd | 0.0028 | 0.008 | 0.005 | 0.006 |
| Nucleotide diversity (Pi) | 0.01168 | 0.01235 | 0.01031 | 0.01135 |
| - Standard deviation of Pi | 0.00021 | 0.00041 | 0.00042 | 0.00035 |
| Theta (per site) from S (Theta-W) | 0.0129 | 0.01232 | 0.01076 | 0.00876 |
| - Standard deviation of theta (no recombination) | 0.00324 | 0.00366 | 0.00323 | 0.00275 |
| - Standard deviation of theta (free recombination) | 0.00192 | 0.00208 | 0.00193 | 0.00175 |
| Theta (per site) from Pi | 0.01186 | 0.01255 | 0.01046 | 0.01152 |
| Average number of nucleotide differences (k) | 6.634 | 7.014 | 5.857 | 6.446 |
| Theta estimated from Eta | 7.497 | 7.197 | 6.113 | 4.974 |
| Fu and Li’s D test statistic | -1.06565 | 0.07787 | 0.37166 | 1.10783 |
| - Statistical significance | NS | NS | NS | NS |
| Fu and Li’s F test statistic | -0.89120 | 0.01977 | 0.21338 | 1.22946 |
| - Statistical significance | NS | NS | NS | NS |
| Tajima’s D | -0.33236 | -0.08044 | -0.08044 | 0.89836 |
| - Statistical significance | NS | NS | NS | NS |
| Synonymous sites Tajima’s D(Syn) | 0.20605 | -0.28416 | -0.28416 | 1.00055 |
| - Statistical significance | NS | NS | NS | NS |
| Nonsynonymous sites Tajima’s D(Nonsyn) | -0.19558 | 0.27317 | 0.27317 | 0.72368 |
| - Statistical significance | NS | NS | NS | NS |
| Silent sites Tajima’s D(Sil) | 0.20605 | -0.28416 | -0.28416 | 1.00055 |
| - Statistical significance | NS | NS | NS | NS |
| Tajima’s D (Nonsyn/Syn) ration | -0.94920 | -0.96133 | -0.96133 | 0.72329 |
| ω (Ka/Ks) | --- | 0.824 | 0.670 | 0.729 |

NS=*p*>0.10; NS^1^=0.10 > *p* > 0.05; *=*p*<0.05
