# Supplementary material for: Phlebotomus papatasi sand fly predicted salivary protein diversity and immune response potential based on in silico prediction in Egypt and Jordan populations
Source: PLoS Negl Trop Dis. 2020 Jul 13;14(7):e0007489. doi: 10.1371/journal.pntd.0007489 (PMC7377520; doi:10.1371/journal.pntd.0007489)
Supplement: S10 Table — (DOCX) [file pntd.0007489.s010.docx]

**S10 Table. *PpSP32* pairwise comparisons of genetic differentiation estimates.**

| POP 1 | POP 2 | Hs | Ks | Gst | Fst | Dxy | Da |
| --- | --- | --- | --- | --- | --- | --- | --- |
| PPAW | PPJM | 0.98298 | 6.41566 | 0.00503 | 0.06816 | 0.01216 | 0.00083 |
| PPAW | PPJS | 0.98206 | 6.72667 | 0.00209 | 0.06508 | 0.01267 | 0.00082 |
| PPJM | PPJS | 0.98392 | 6.14514 | 0.00188 | 0.00140 | 0.01085 | 0.00002 |
