# Supplementary material for: Phlebotomus papatasi sand fly predicted salivary protein diversity and immune response potential based on in silico prediction in Egypt and Jordan populations
Source: PLoS Negl Trop Dis. 2020 Jul 13;14(7):e0007489. doi: 10.1371/journal.pntd.0007489 (PMC7377520; doi:10.1371/journal.pntd.0007489)
Supplement: S11 Table — Ka/Ks were plotted for every 70 codons. Values greater than one suggest the potential for positive selection. ----indicates a lack of polymorphic data in the window to calculate a Ka/Ks value. (DOCX) [file pntd.0007489.s011.docx]

**S11 Table. PpSP32 sliding window analysis.**

|  | Ka/Ks | | |
| --- | --- | --- | --- |
| Sliding Window | PPAW | PPJM | PPJS |
| 1-70 | 0.011 | 0.000 | 0.000 |
| 71-140 | 6.043 | 1.573 | 1.255 |
| 141-210 | 0.000 | 0.000 | 0.000 |
| 211-280 | 0.827 | 1.046 | --- |
| 281-350 | --- | --- | --- |
| 351-420 | 0.052 | 0.000 | 0.295 |
| 421-490 | 0.564 | 0.477 | 0.648 |
| 491-560 | --- | --- | --- |
| 561-568 | 0.000 | 0.000 | 0.000 |

Ka/Ks were plotted for every 70 codons. Values greater than one suggest the potential for positive selection. ---- indicates a lack of polymorphic data in the window to calculate a Ka/Ks value.
