# Supplementary material for: Phlebotomus papatasi sand fly predicted salivary protein diversity and immune response potential based on in silico prediction in Egypt and Jordan populations
Source: PLoS Negl Trop Dis. 2020 Jul 13;14(7):e0007489. doi: 10.1371/journal.pntd.0007489 (PMC7377520; doi:10.1371/journal.pntd.0007489)
Supplement: S12 Table — (DOCX) [file pntd.0007489.s012.docx]

**S12 Table**. **PpSP36 population genetics analyses for *P. papatasi* populations.**

| Population | All Data | PPAW | PPJM | PPJS |
| --- | --- | --- | --- | --- |
| Number of Sequences | 82 | 25 | 22 | 35 |
| Number of Sites | 637 | 637 | 637 | 637 |
| - Monomorphic | 590 | 601 | 612 | 602 |
| - Polymorphic | 47 | 36 | 25 | 35 |
| Singleton variable sites | 14 | 13 | 4 | 9 |
| - Site positions | 11, 137, 182, 221, 293, 353, 388, 434, 459, 476, 505, 509, 564, 617 | 11, 137, 173, 182, 293, 353, 427, 428, 434, 485, 505, 527, 564 | 120, 342, 362, 499 | 83, 173, 221, 296, 388, 459, 476, 509, 617 |
| Parsimony informative sites | 33 | 23 | 21 | 26 |
| - Site positions | 17, 72, 74, 83, 120, 146, 155, 173, 197, 258, 296, 323, 329, 342, 350, 359, 362, 383, 386, 427, 428, 437, 461, 470, 485, 499, 521, 527, 563, 565, 623, 626, 635 | 17, 72, 74, 120, 146, 155, 197, 296, 323, 342, 359, 362, 383, 386, 437, 461, 470, 499, 521, 563, 565, 623, 626 | 72, 74, 83, 146, 296, 323, 329, 359, 386, 427, 428, 437, 461, 470, 485, 521, 527, 563, 565, 623, 635 | 17, 72, 74, 120, 146, 258, 329, 342, 350, 359, 383, 386, 427, 437, 461, 470, 485, 499, 521, 527, 563, 565, 623, 635 |
| Segregating sites (S) | 47 | 36 | 25 | 35 |
| Total number of mutations (Eta) | 50 | 36 | 26 | 37 |
| Total number of synonymous changes | 35 | 27 | 20 | 24 |
| - Site positions | 11, 17, 72, 74, 83, 137, 146, 155, 173, 182, 197, 221, 293, 296, 323, 329, 350, 353, 359, 362, 386, 434, 437, 461, 476, 485, 509, 521, 527, 563, 563, 564, 617, 623, 635 | 11, 17, 72, 74, 137, 146, 155, 173, 182, 197, 293, 296, 323, 353, 359, 362, 386, 428, 434, 437, 461, 485, 521, 527, 563, 564, 623 | 72, 74, 83, 146, 296, 323, 329, 359, 362, 386, 428, 437, 461, 485, 521, 527, 563, 563, 623, 635 | 17, 72, 74, 83, 146, 173, 221, 296, 323, 329, 350, 359, 386, 437, 461, 476, 485, 509, 521, 527, 563, 617, 623, 635 |
| Total number of replacement changes | 12 | 9 | 6 | 10 |
| - Site positions | 120, 258, 323, 342, 383, 388, 459, 470, 499, 505, 565, 626 | 120, 342, 383, 427, 470, 499, 505, 565, 626 | 120, 342, 427, 470, 499, 565 | 120, 258, 323, 342, 383, 388, 459, 470, 499, 565 |
| Number of haplotypes | 110 | 41 | 27 | 53 |
| Haplotype diversity (Hd) | 0.9798 | 0.987 | 0.923 | 0.984 |
| - Standard deviation of Hd | 0.0060 | 0.008 | 0.034 | 0.007 |
| Nucleotide diversity (Pi) | 0.01040 | 0.00939 | 0.00862 | 0.01076 |
| - Standard deviation of Pi | 0.00034 | 0.00063 | 0.00076 | 0.00053 |
| Theta (per site) from S (Theta-W) | 0.01300 | 0.01262 | 0.00902 | 0.01140 |
| - Standard deviation of theta (no recombination) | 0.00341 | 0.00400 | 0.00307 | 0.00346 |
| - Standard deviation of theta (free recombination) | 0.0019 | 0.00210 | 0.00180 | 0.00193 |
| Theta (per site) from Pi | 0.01054 | 0.00951 | 0.00872 | 0.01091 |
| Average number of nucleotide differences (k) | 6.623 | 5.983 | 5.494 | 6.852 |
| Theta estimated from Eta | 8.812 | 8.037 | 5.977 | 7.679 |
| Fu and Li’s D test statistic | -1.58309 | -1.10475 | 0.60959 | -0.55962 |
| - Statistical significance | NS | NS | NS | NS |
| Fu and Li’s F test statistic | -1.47242 | -1.21234 | 0.36300 | -0.57340 |
| - Statistical significance | NS | NS | NS | NS |
| Tajima’s D | -0.75170 | -0.86327 | -0.27071 | -0.34842 |
| - Statistical significance | NS | NS | NS | NS |
| Synonymous sites Tajima’s D(Syn) | -0.75939 | -0.84923 | -0.23532 | -0.27931 |
| - Statistical significance | NS | NS | NS | NS |
| Nonsynonymous sites Tajima’s D(Nonsyn) | -0.65340 | -0.69752 | -0.28615 | -0.49815 |
| - Statistical significance | NS | NS | NS | NS |
| Silent sites Tajima’s D(Sil) | -0.75939 | -0.84923 | -0.23532 | -0.27931 |
| - Statistical significance | NS | NS | NS | NS |
| Tajima’s D (Nonsyn/Syn) ration | 0.86042 | 0.82136 | 1.21601 | 1.78351 |
| ω (Ka/Ks) | --- | 0.086 | 0.072 | 0.102 |

NS=*p*>0.10; NS^1^=0.10 > *p* > 0.05; *=*p*<0.05
