# Supplementary material for: Phlebotomus papatasi sand fly predicted salivary protein diversity and immune response potential based on in silico prediction in Egypt and Jordan populations
Source: PLoS Negl Trop Dis. 2020 Jul 13;14(7):e0007489. doi: 10.1371/journal.pntd.0007489 (PMC7377520; doi:10.1371/journal.pntd.0007489)
Supplement: S13 Table — (DOCX) [file pntd.0007489.s013.docx]

**S13 Table. *PpSP36* pairwise comparisons of genetic differentiation estimates.**

| POP 1 | POP 2 | Hs | Ks | Gst | Fst | Dxy | Da |
| --- | --- | --- | --- | --- | --- | --- | --- |
| PPAW | PPJM | 0.95702 | 5.75387 | 0.01593 | 0.17844 | 0.01096 | 0.00196 |
| PPAW | PPJS | 0.98513 | 6.48996 | 0.00460 | 0.06784 | 0.01081 | 0.00073 |
| PPJM | PPJS | 0.96055 | 6.32783 | 0.00885 | 0.04885 | 0.01019 | 0.00050 |
