# Supplementary material for: Phlebotomus papatasi sand fly predicted salivary protein diversity and immune response potential based on in silico prediction in Egypt and Jordan populations
Source: PLoS Negl Trop Dis. 2020 Jul 13;14(7):e0007489. doi: 10.1371/journal.pntd.0007489 (PMC7377520; doi:10.1371/journal.pntd.0007489)
Supplement: S14 Table — Ka/Ks were plotted for every 70 codons. Values greater than one suggest the potential for positive selection. ----indicates a lack of polymorphic data in the window to calculate a Ka/Ks value. (DOCX) [file pntd.0007489.s014.docx]

**S14 Table. PpSP36 sliding window analysis.**

|  | Ka/Ks | | |
| --- | --- | --- | --- |
| Sliding Window | PPAW | PPJM | PPJS |
| 1-72 | 0.000 | 0.000 | 0.000 |
| 72-141 | 0.047 | 0.014 | 0.040 |
| 142-211 | 0.000 | 0.000 | 0.000 |
| 212-281 | 0.000 | 0.000 | 0.934 |
| 282-351 | 0.067 | 0.022 | 0.075 |
| 352-421 | 0.043 | 0.000 | 0.076 |
| 422-491 | 0.065 | 0.144 | 0.167 |
| 492-561 | 0.082 | 0.024 | 0.089 |
| 562-631 | 0.337 | 0.401 | 0.369 |
| 632-637 | 0.000 | 0.000 | 0.000 |

Ka/Ks were plotted for every 70 codons. Values greater than one suggest the potential for positive selection. ---- indicates a lack of polymorphic data in the window to calculate a Ka/Ks value.
