# Supplementary material for: Phlebotomus papatasi sand fly predicted salivary protein diversity and immune response potential based on in silico prediction in Egypt and Jordan populations
Source: PLoS Negl Trop Dis. 2020 Jul 13;14(7):e0007489. doi: 10.1371/journal.pntd.0007489 (PMC7377520; doi:10.1371/journal.pntd.0007489)
Supplement: S15 Table — (DOCX) [file pntd.0007489.s015.docx]

**S15 Table**. **PpSP42 population genetics analyses for *P. papatasi* populations**

| Population | All Data | PPAW | PPJM | PPJS |
| --- | --- | --- | --- | --- |
| Number of Sequences | 109 | 27 | 45 | 37 |
| Number of Sites | 614 | 614 | 614 | 614 |
| - Monomorphic | 561 | 574 | 572 | 577 |
| - Polymorphic | 53 | 40 | 42 | 37 |
| Singleton variable sites | 9 | 5 | 3 | 3 |
| - Site positions | 5, 7, 127, 128, 311, 378, 479, 524, 559 | 5, 7, 127, 128, 559 | 311, 312, 524 | 321, 378, 479 |
| Parsimony informative sites | 44 | 35 | 39 | 34 |
| - Site positions | 33, 58, 63, 88, 98, 105, 116, 123, 131, 134, 221, 223, 226, 230, 233, 241, 252, 290, 296, 301, 302, 309, 312, 321, 341, 344, 382, 384, 388, 391, 398, 401, 416, 431, 460, 473, 476, 482, 488, 494, 530, 551, 570, 596 | 33, 63, 88, 98, 116, 123, 131, 223, 233, 241, 252, 296, 301, 302, 309, 312, 321, 341, 344, 382, 384, 388, 391, 398, 401, 416, 431, 473, 476, 488, 494, 530, 551, 570, 596 | 58, 63, 88, 116, 123, 131, 134, 221, 223, 226, 230, 233, 241, 252, 290, 296, 301, 302, 309, 321, 341, 344, 382, 388, 391, 398, 401, 416, 431, 460, 473, 476, 482, 488, 494, 530, 551, 570, 596 | 58, 63, 88, 105, 116, 123, 131, 223, 233, 241, 252, 296, 301, 302, 309, 341, 344, 382, 384, 388, 391, 398, 401, 416, 431, 473, 476 |
| Segregating sites (S) | 53 | 40 | 42 | 37 |
| Total number of mutations (Eta) | 55 | 41 | 43 | 37 |
| Total number of synonymous changes | 28 | 22 | 24 | 20 |
| - Site positions | 5, 98, 116, 128, 131, 134, 221, 233, 290, 296, 311, 321, 341, 344, 398, 401, 416, 431, 473, 476, 479, 482, 488, 494, 524, 530, 551, 596 | 5, 98, 116, 128, 131, 233, 296, 302, 321, 341, 344, 398, 401, 416, 431, 473, 476, 488, 494, 530, 551, 596 | 116, 131, 134, 221, 233, 290, 296, 311, 321, 341, 344, 398, 401, 416, 431, 473, 476, 482, 488, 494, 524, 530, 551, 596 | 116, 131, 233, 296, 321, 341, 344, 398, 401, 416, 431, 473, 476, 479, 482, 488, 494, 530, 551, 596 |
| Total number of replacement changes | 24 | 19 | 19 | 17 |
| - Site positions | 7, 33, 58, 63, 88, 105, 123, 127, 223, 223, 226, 230, 241, 252, 309, 312, 378, 382, 384, 388, 391, 460, 559, 570 | 7, 33, 63, 88, 123, 127, 223, 241, 252, 301, 302, 309, 312, 382, 384, 388, 391, 559, 570 | 58, 63, 88, 123, 223, 223, 226, 230, 241, 252, 301, 302, 309, 312, 382, 388, 391, 460, 570 | 58, 63, 88, 105, 123, 223, 241, 252, 301, 302, 309, 378, 382, 384, 388, 391, 570 |
| Number of haplotypes | 133 | 38 | 60 | 51 |
| Haplotype diversity (Hd) | 0.9878 | 0.987 | 0.985 | 0.983 |
| - Standard deviation of Hd | 0.0032 | 0.006 | 0.005 | 0.007 |
| Nucleotide diversity (Pi) | 0.01921 | 0.01992 | 0.01950 | 0.01764 |
| - Standard deviation of Pi | 0.0032 | 0.00085 | 0.00086 | 0.00110 |
| Theta (per site) from S (Theta-W) | 0.01448 | 0.01430 | 0.01349 | 0.01236 |
| - Standard deviation of theta (no recombination) | 0.00361 | 0.00443 | 0.00387 | 0.00370 |
| - Standard deviation of theta (free recombination) | 0.00199 | 0.00226 | 0.00208 | 0.00203 |
| Theta (per site) from Pi | 0.01971 | 0.02047 | 0.02002 | 0.01806 |
| Average number of nucleotide differences (k) | 11.793 | 12.233 | 11.974 | 10.831 |
| Theta estimated from Eta | 9.229 | 8.997 | 8.479 | 7.591 |
| Fu and Li’s D test statistic | 0.07023 | 0.88429 | 1.35699 | 1.21069 |
| - Statistical significance | NS | NS | NS 0.10 > P > 0.05 | NS |
| Fu and Li’s F test statistic | 0.49007 | 1.19815 | 1.60441 | 1.52186 |
| - Statistical significance | NS | NS | NS 0.10 > P > 0.05 | NS |
| Tajima’s D | 0.82581 | 1.21311 | 1.31080 | 1.37241 |
| - Statistical significance | NS | NS | NS | NS |
| Synonymous sites Tajima’s D(Syn) | 0.97682 | 1.39270 | 1.11438 | 1.28662 |
| - Statistical significance | NS | NS | NS | NS |
| Nonsynonymous sites Tajima’s D(Nonsyn) | 0.39263 | 1.22137 | 1.35304 | 1.2550 |
| - Statistical significance | NS | NS | NS | NS |
| Silent sites Tajima’s D(Sil) | 0.97682 | 1.39270 | 1.11438 | 1.28662 |
| - Statistical significance | NS | NS | NS | NS |
| Tajima’s D (Nonsyn/Syn) ration | 0.40195 | 0.87698 | 1.21417 | 0.97545 |
| ω (Ka/Ks) | --- | 0.269 | 0.253 | 0.255 |

NS=*p*>0.10; NS^1^=0.10 > *p* > 0.05; *=*p*<0.05
