# Supplementary material for: Phlebotomus papatasi sand fly predicted salivary protein diversity and immune response potential based on in silico prediction in Egypt and Jordan populations
Source: PLoS Negl Trop Dis. 2020 Jul 13;14(7):e0007489. doi: 10.1371/journal.pntd.0007489 (PMC7377520; doi:10.1371/journal.pntd.0007489)
Supplement: S16 Table — (DOCX) [file pntd.0007489.s016.docx]

**S16 Table. *PpSP42* pairwise comparisons of genetic differentiation estimates.**

| POP 1 | POP 2 | Hs | Ks | Gst | Fst | Dxy | Da |
| --- | --- | --- | --- | --- | --- | --- | --- |
| PPAW | PPJM | 0.98534 | 12.07119 | 0.00328 | 0.00610 | 0.01983 | 0.00012 |
| PPAW | PPJS | 0.98476 | 11.42223 | 0.00264 | 0.02676 | 0.01930 | 0.00052 |
| PPJM | PPJS | 0.98399 | 11.45832 | 0.00202 | 0.02256 | 0.01900 | 0.00043 |
