# Supplementary material for: Phlebotomus papatasi sand fly predicted salivary protein diversity and immune response potential based on in silico prediction in Egypt and Jordan populations
Source: PLoS Negl Trop Dis. 2020 Jul 13;14(7):e0007489. doi: 10.1371/journal.pntd.0007489 (PMC7377520; doi:10.1371/journal.pntd.0007489)
Supplement: S17 Table — Ka/Ks were plotted for every 70 codons. Values greater than one suggest the potential for positive selection. ----indicates a lack of polymorphic data in the window to calculate a Ka/Ks value. (DOCX) [file pntd.0007489.s017.docx]

**S17 Table. PpSP42 sliding window analysis.**

|  | Ka/Ks | | |
| --- | --- | --- | --- |
| Sliding Window | PPAW | PPJM | PPJS |
| 1-72 | 2.339 | --- | --- |
| 72-141 | 0.300 | 0.294 | 0.386 |
| 142-211 | 0.000 | 0.000 | 0.000 |
| 212-281 | 0.825 | 0.809 | 0.776 |
| 282-351 | 0.216 | 0.196 | 0.218 |
| 352-421 | 0.443 | 0.439 | 0.439 |
| 422-491 | 0.000 | 0.037 | 0.000 |
| 492-561 | 0.010 | 0.000 | 0.000 |
| 562-614 | 0.203 | 0.313 | 0.313 |

Ka/Ks were plotted for every 70 codons. Values greater than one suggest the potential for positive selection. ---- indicates a lack of polymorphic data in the window to calculate a Ka/Ks value.
