# Supplementary material for: Phlebotomus papatasi sand fly predicted salivary protein diversity and immune response potential based on in silico prediction in Egypt and Jordan populations
Source: PLoS Negl Trop Dis. 2020 Jul 13;14(7):e0007489. doi: 10.1371/journal.pntd.0007489 (PMC7377520; doi:10.1371/journal.pntd.0007489)
Supplement: S18 Table — (DOCX) [file pntd.0007489.s018.docx]

**S18 Table**. **PpSP44 population genetics analyses for *P. papatasi* populations.**

| Population | All Data | PPAW | PPJM | PPJS |
| --- | --- | --- | --- | --- |
| Number of Sequences | 121 | 35 | 44 | 42 |
| Number of Sites | 675 | 675 | 675 | 675 |
| - Monomorphic | 639 | 661 | 648 | 654 |
| - Polymorphic | 36 | 14 | 27 | 21 |
| Singleton variable sites | 12 | 3 | 7 | 3 |
| - Site positions | 30, 40, 81, 234, 241, 254, 281, 282, 427, 530, 622, 638 | 81, 132, 530 | 40, 234, 241, 254, 281, 282, 427 | 30, 622, 638 |
| Parsimony informative sites | 24 | 11 | 20 | 18 |
| - Site positions | 52, 57, 78, 116, 127, 132, 157, 187, 202, 205, 233, 272, 300, 331, 343, 364, 409, 478, 547, 558, 586, 595, 608, 659 | 78, 127, 157, 202, 205, 233, 300, 547, 586, 608, 659 | 57, 78, 116, 127, 132, 157, 187, 202, 205, 233, 272, 300, 331, 343, 364, 409, 478, 586, 608, 659 | 52, 78, 116, 132, 157, 187, 202, 205, 272, 300, 331, 343, 364, 558, 586, 595, 608, 659 |
| Segregating sites (S) | 36 | 14 | 27 | 21 |
| Total number of mutations (Eta) | 38 | 14 | 29 | 22 |
| Total number of synonymous changes | 18 | 5 | 14 | 10 |
| - Site positions | 40, 52, 127, 157, 187, 202, 202, 241, 331, 343, 364, 409, 427, 478, 547, 586, 595, 622 | 127, 157, 202, 547, 586 | 40, 127, 157, 187, 202, 202, 241, 331, 343, 364, 409, 427, 478, 586 | 52, 157, 187, 202, 331, 343, 364, 586, 595, 622 |
| Total number of replacement changes | 20 | 8 | 15 | 12 |
| - Site positions | 30, 57, 78, 81, 116, 132, 205, 233, 234, 254, 272, 281, 282, 300, 364, 530, 558, 608, 638, 659 | 78, 81, 132, 205, 233, 300, 530, 608, 659 | 57, 78, 116, 132, 205, 233, 234, 254, 272, 281, 282, 300, 364, 608, 659 | 30, 78, 116, 132, 205, 272, 300, 364, 558, 608, 638, 659 |
| Number of haplotypes | 65 | 21 | 35 | 33 |
| Haplotype diversity (Hd) | 0.907 | 0.828 | 0.943 | 0.904 |
| - Standard deviation of Hd | 0.015 | 0.040 | 0.015 | 0.024 |
| Nucleotide diversity (Pi) | 0.00429 | 0.00456 | 0.00439 | 0.00346 |
| - Standard deviation of Pi | 0.00022 | 0.00035 | 0.00034 | 0.00030 |
| Theta (per site) from S (Theta-W) | 0.00879 | 0.00430 | 0.00792 | 0.00622 |
| - Standard deviation of theta (no recombination) | 0.00232 | 0.00157 | 0.00244 | 0.00202 |
| - Standard deviation of theta (free recombination) | 0.00147 | 0.00115 | 0.00152 | 0.00136 |
| Theta (per site) from Pi | 0.00432 | 0.00459 | 0.00442 | 0.00347 |
| Average number of nucleotide differences (k) | 2.898 | 3.077 | 2.963 | 2.334 |
| Theta estimated from Eta | 6.266 | 2.905 | 5.744 | 4.398 |
| Fu and Li’s D test statistic | -1.97128 | -0.02758 | -0.39141 | 0.57868 |
| - Statistical significance | NS 0.10 > P > 0.05 | NS | NS | NS |
| Fu and Li’s F test statistic | -2.14766 | 0.04949 | -0.97154 | -0.19067 |
| - Statistical significance | NS 0.10 > P > 0.05 | NS | NS | NS |
| Tajima’s D | -1.53419 | 0.17028 | -1.4897 | -1.40699 |
| - Statistical significance | NS 0.10 > P > 0.05 | NS | NS | NS |
| Synonymous sites Tajima’s D(Syn) | -1.58611 | 0.05434 | -1.42572 | -1.48272 |
| - Statistical significance | NS 0.10 > P > 0.05 | NS | NS | NS |
| Nonsynonymous sites Tajima’s D(Nonsyn) | -1.14671 | 0.20787 | -1.14585 | -0.90502 |
| - Statistical significance | NS | NS | NS | NS |
| Silent sites Tajima’s D(Sil) | -1.58611 | 0.05434 | -1.42572 | -1.48272 |
| - Statistical significance | NS 0.10 > P > 0.05 | NS | NS | NS |
| Tajima’s D (Nonsyn/Syn) ration | 0.72297 | 3.82514 | 0.80370 | 0.61038 |
| ω (Ka/Ks) | --- | 0.582 | 0.388 | 0.564 |

NS=*p*>0.10; NS^1^=0.10 > *p* > 0.05; *=*p*<0.05
