# Supplementary material for: Phlebotomus papatasi sand fly predicted salivary protein diversity and immune response potential based on in silico prediction in Egypt and Jordan populations
Source: PLoS Negl Trop Dis. 2020 Jul 13;14(7):e0007489. doi: 10.1371/journal.pntd.0007489 (PMC7377520; doi:10.1371/journal.pntd.0007489)
Supplement: S19 Table — (DOCX) [file pntd.0007489.s019.docx]

**S19 Table. *PpSP44* pairwise comparisons of genetic differentiation estimates.**

| POP 1 | POP 2 | Hs | Ks | Gst | Fst | Dxy | Da |
| --- | --- | --- | --- | --- | --- | --- | --- |
| PPAW | PPJM | 0.89214 | 3.01394 | 0.01755 | 0.09062 | 0.00492 | 0.00045 |
| PPAW | PPJS | 0.86938 | 2.67203 | 0.01213 | 0.09323 | 0.00442 | 0.00041 |
| PPJM | PPJS | 0.92394 | 2.65613 | -0.00039 | -0.00020 | 0.00392 | 0.00000 |
