# Supplementary material for: Phlebotomus papatasi sand fly predicted salivary protein diversity and immune response potential based on in silico prediction in Egypt and Jordan populations
Source: PLoS Negl Trop Dis. 2020 Jul 13;14(7):e0007489. doi: 10.1371/journal.pntd.0007489 (PMC7377520; doi:10.1371/journal.pntd.0007489)
Supplement: S20 Table — Ka/Ks were plotted for every 70 codons. Values greater than one suggest the potential for positive selection. ----indicates a lack of polymorphic data in the window to calculate a Ka/Ks value. (DOCX) [file pntd.0007489.s020.docx]

**S20 Table. PpSP44 sliding window analysis.**

|  | Ka/Ks | | |
| --- | --- | --- | --- |
| Sliding Window | PPAW | PPJM | PPJS |
| 1-7 | 0.000 | 0.754 | 0.193 |
| 71-140 | 0.274 | 2.293 | --- |
| 141-210 | 0.344 | 0.103 | 0.125 |
| 211-280 | --- | 1.961 | --- |
| 281-350 | --- | 0.383 | 0.283 |
| 351-420 | 0.000 | 0.048 | 0.153 |
| 421-490 | 0.000 | 0.000 | 0.000 |
| 491-560 | 0.054 | 0.000 | --- |
| 561-630 | 0.865 | 0.438 | 0.243 |
| 631-675 | --- | --- | --- |

Ka/Ks were plotted for every 70 codons. Values greater than one suggest the potential for positive selection. ---- indicates a lack of polymorphic data in the window to calculate a Ka/Ks value.
