# Supplementary material for: Phlebotomus papatasi sand fly predicted salivary protein diversity and immune response potential based on in silico prediction in Egypt and Jordan populations
Source: PLoS Negl Trop Dis. 2020 Jul 13;14(7):e0007489. doi: 10.1371/journal.pntd.0007489 (PMC7377520; doi:10.1371/journal.pntd.0007489)
Supplement: S21 Table — NS = p>0.10; NS1 = 0.10 > p > 0.05; * = p<0.05. (DOCX) [file pntd.0007489.s021.docx]

**S21 Table. Summary Tajima’s D and Ka/Ks analysis for all *P. papatasi* salivary proteins studied.**

| Salivary Protein | Tajima’s D | Tajima’s D  Significance | Ka/Ks | | |
| --- | --- | --- | --- | --- | --- |
|  | All Populations | | PPAW | PPJM | PPJS |
| PpSP12 | 0.33033 | NS | 0.222 | 0.284 | 0.242 |
| PpSP14 | -1.33534 | NS | 0.877 | 0.879 | 1.242 |
| PpSP28 | 0.38514 | NS | 0.480 | 0.487 | 0.497 |
| PpSP29 | -0.70794 | NS | 0.066 | 0.082 | 0.080 |
| PpSP30 | -1.22339 | NS | 0.095 | 0.190 | 0.166 |
| PpSP32 | -0.33236 | NS | 0.824 | 0.670 | 0.729 |
| PpSP36 | -0.75170 | NS | 0.086 | 0.072 | 0.102 |
| PpSP42 | 0.82581 | NS | 0.269 | 0.253 | 0.255 |
| PpSP44 | -1.53419 | NS^1^ | 0.582 | 0.388 | 0.564 |

NS=*p*>0.10; NS^1^=0.10 > *p* > 0.05; *=*p*<0.05
