# Supplementary material for: Phlebotomus papatasi sand fly predicted salivary protein diversity and immune response potential based on in silico prediction in Egypt and Jordan populations
Source: PLoS Negl Trop Dis. 2020 Jul 13;14(7):e0007489. doi: 10.1371/journal.pntd.0007489 (PMC7377520; doi:10.1371/journal.pntd.0007489)
Supplement: S13 Fig — (A) Weblogo illustrating the relative frequencies of nucleotide polymorphisms in wild caught P. papatasi populations from PPAW, PPJM, and PPJS. (B) Weblogo illustrating the relative frequencies of amino acid polymorphisms in wild caught P. papatasi populations from PPAW, PPJM, and PPJS. (PPTX) [file pntd.0007489.s034.pptx]

## Slide 1
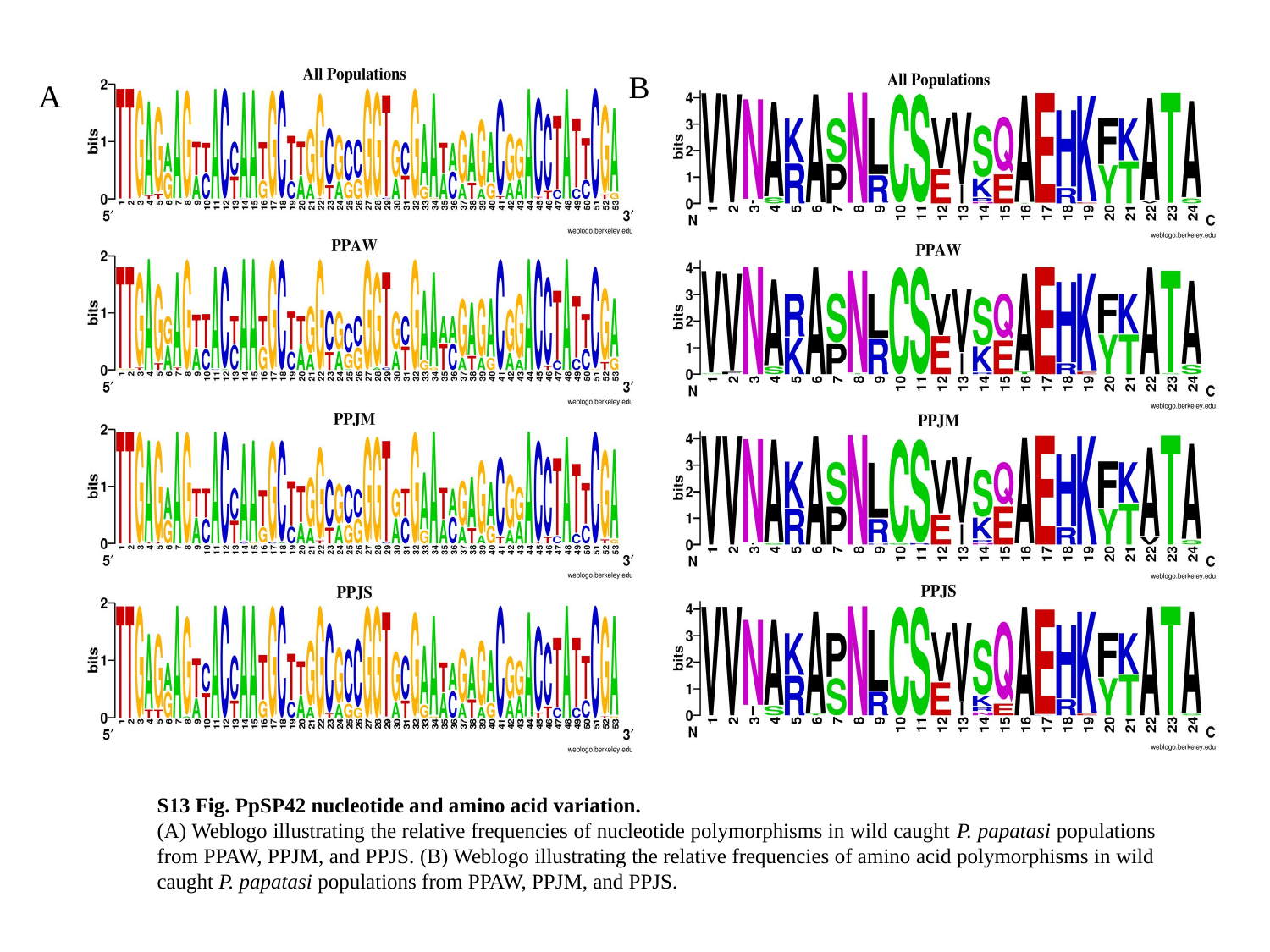

B
A
S13 Fig. PpSP42 nucleotide and amino acid variation.
(A) Weblogo illustrating the relative frequencies of nucleotide polymorphisms in wild caught P. papatasi populations from PPAW, PPJM, and PPJS. (B) Weblogo illustrating the relative frequencies of amino acid polymorphisms in wild caught P. papatasi populations from PPAW, PPJM, and PPJS.
