# Supplementary material for: Phlebotomus papatasi sand fly predicted salivary protein diversity and immune response potential based on in silico prediction in Egypt and Jordan populations
Source: PLoS Negl Trop Dis. 2020 Jul 13;14(7):e0007489. doi: 10.1371/journal.pntd.0007489 (PMC7377520; doi:10.1371/journal.pntd.0007489)
Supplement: S15 Fig — The mature PpSP42 amino acid sequence predicted secondary structure. Yellow highlighted amino acids indicate the predicted MHC class II predicted promiscuous peptides. Individual amino acids underlined in black indicate unique polymorphic sites. Predicted secondary structure based on sequence accession #AGE83094 [51]. (PPTX) [file pntd.0007489.s036.pptx]

## Slide 1
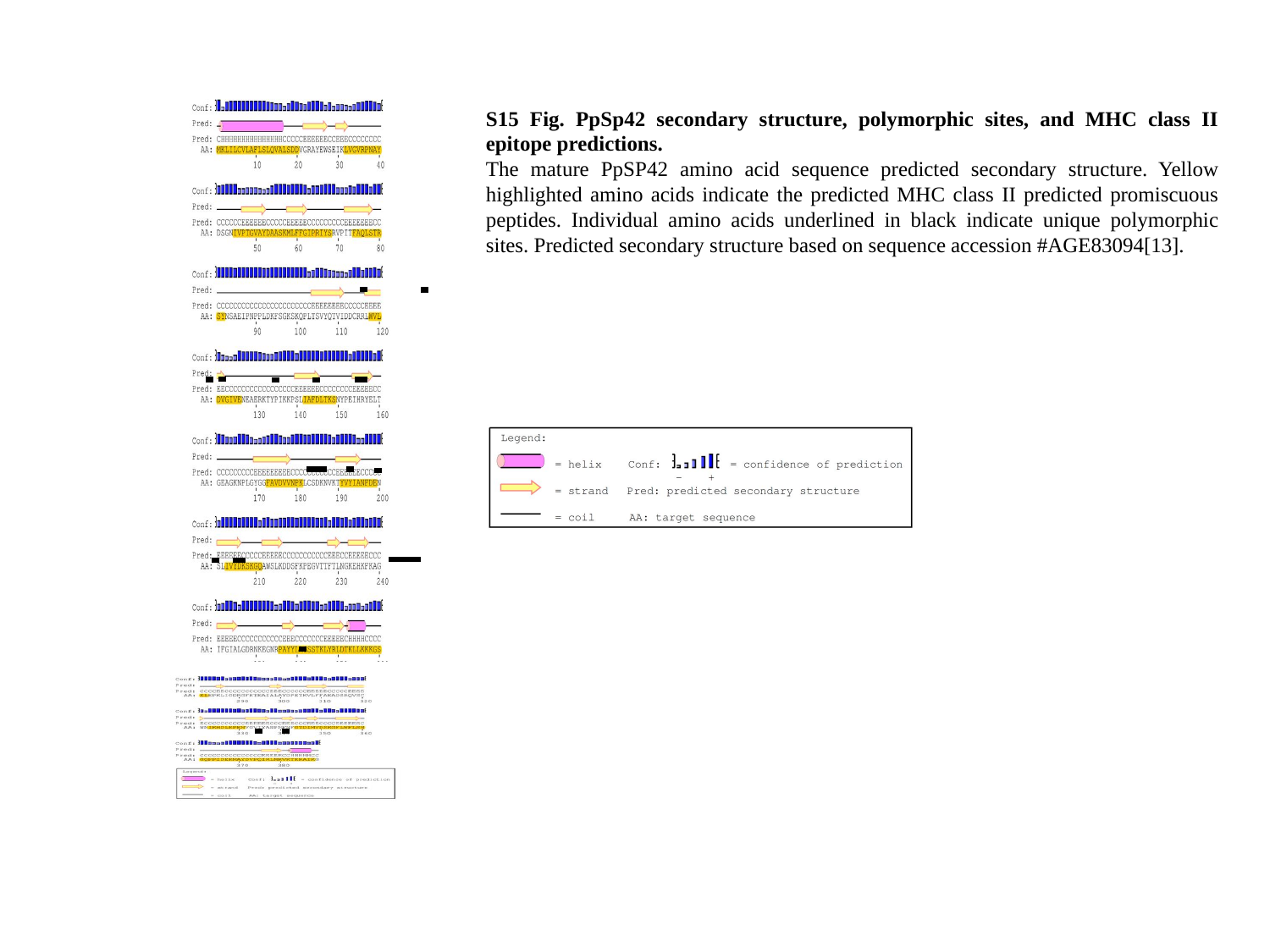

S15 Fig. PpSp42 secondary structure, polymorphic sites, and MHC class II epitope predictions.
The mature PpSP42 amino acid sequence predicted secondary structure. Yellow highlighted amino acids indicate the predicted MHC class II predicted promiscuous peptides. Individual amino acids underlined in black indicate unique polymorphic sites. Predicted secondary structure based on sequence accession #AGE83094[13].
