# Supplementary material for: Phlebotomus papatasi sand fly predicted salivary protein diversity and immune response potential based on in silico prediction in Egypt and Jordan populations
Source: PLoS Negl Trop Dis. 2020 Jul 13;14(7):e0007489. doi: 10.1371/journal.pntd.0007489 (PMC7377520; doi:10.1371/journal.pntd.0007489)
Supplement: S17 Fig — Circle size and circle color indicates frequency and geographical location of haplotypes, respectively. Haplotype numbers are indicated next to the corresponding circle H_XX. Red numbers between haplotypes indicate number of mutations between haplotypes. (PPTX) [file pntd.0007489.s038.pptx]

## Slide 1
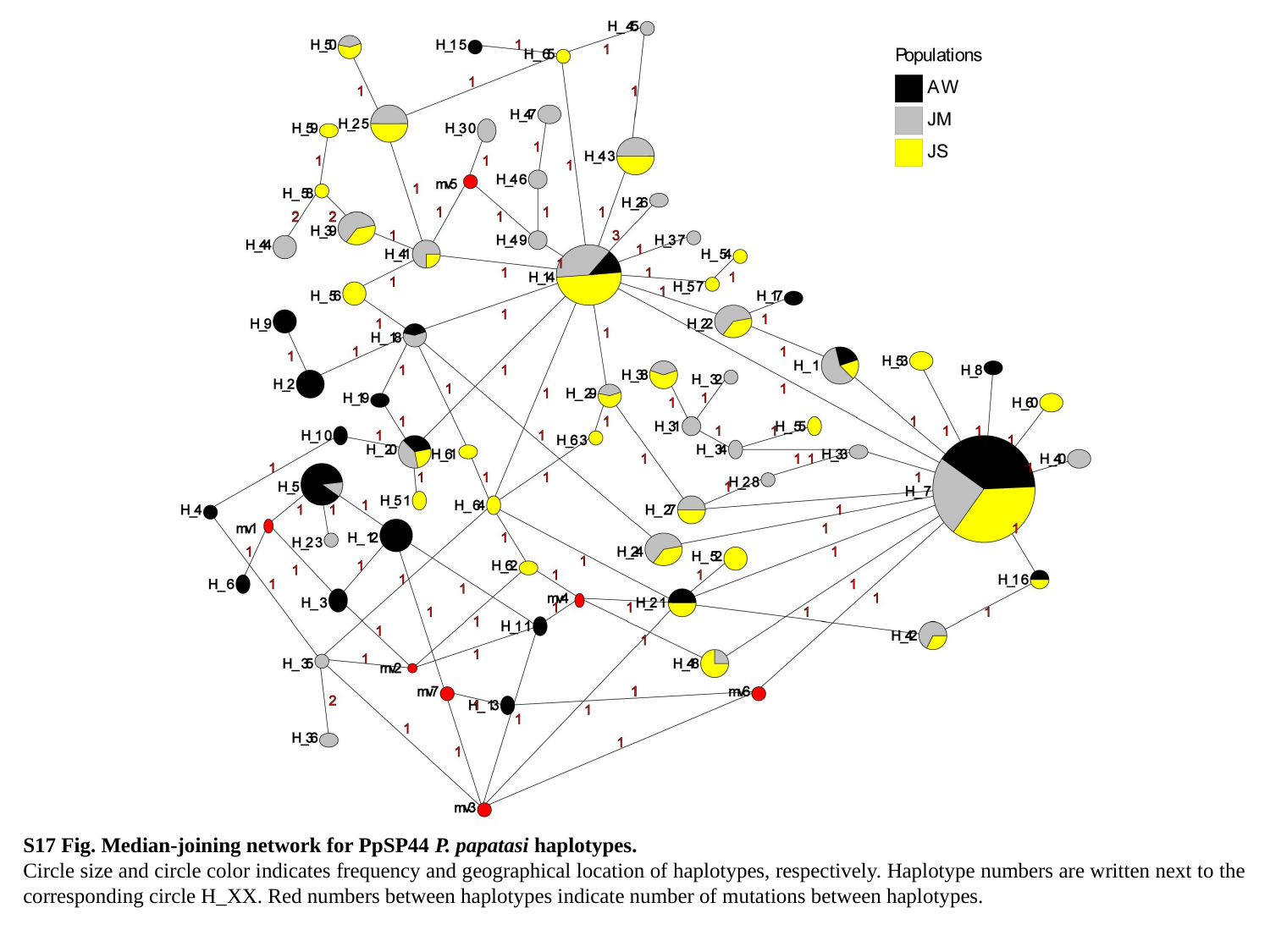

S17 Fig. Median-joining network for PpSP44 P. papatasi haplotypes.
Circle size and circle color indicates frequency and geographical location of haplotypes, respectively. Haplotype numbers are written next to the corresponding circle H_XX. Red numbers between haplotypes indicate number of mutations between haplotypes.
